# Retrograde transport of neurotrophin receptor TrkB-FL induced by excitotoxicity regulates Golgi stability and is a target for stroke neuroprotection

**DOI:** 10.1038/s41419-025-07990-6

**Published:** 2025-08-29

**Authors:** Gema María Esteban-Ortega, Elena Torres-Campos, Margarita Díaz-Guerra

**Affiliations:** 1 Instituto de Investigaciones Biomédicas Sols-Morreale (IIBM), Consejo Superior de Investigaciones Científicas-Universidad Autónoma de Madrid, Madrid, 28029 Spain; 2 Present Address: Centro de Biología Molecular Severo Ochoa (CBMSO), Consejo Superior de Investigaciones Científicas-Universidad Autónoma de Madrid, Nicolás Cabrera,1, Madrid, 28049 Spain

**Keywords:** Stroke, Stroke

## Abstract

Excitotoxicity, aberrant function of survival pathways dependent on brain-derived neurotrophic factor (BDNF), and disruption of the Golgi complex are shared pathological hallmarks in relevant neurological diseases, including stroke. However, the precise interdependence among these mechanisms is not completely defined, knowledge essential for developing neuroprotective strategies. For ischemic stroke, a leading cause of death, disability, and dementia, interfering with excitotoxicity—the major mechanism of neuronal death in the penumbra area—has shown promising results. We are exploring neuroprotection by promoting survival cascades dependent on the BDNF receptor, full-length tropomyosin-related kinase B (TrkB-FL), as these pathways become aberrant after excitotoxicity. We previously developed MTFL_457_, a blood-brain barrier (BBB) permeable neuroprotective peptide containing a TrkB-FL sequence, which efficiently prevents excitotoxicity-induced receptor processing and preserves BDNF-dependent pathways in an ischemia model, where it decreases infarct size and improves neurological outcome. In this work, using cellular and animal models, we demonstrate that excitotoxicity-induced TrkB-FL downregulation is secondary to receptor endocytosis, interaction with the endosomal protein hepatocyte growth factor-regulated tyrosine kinase substrate (Hrs), retrograde transport to the Golgi, and subsequent disruption of this organelle. Interestingly, peptide MTFL_457_ interferes with the TrkB-FL/Hrs interaction and receptor trafficking—processes required for excitotoxic Golgi fragmentation and TrkB-FL cleavage—demonstrating a central role for TrkB-FL in controlling Golgi stability. These results suggest the potential for peptide MTFL_457_ to preserve the function of this organelle and critical neuronal survival pathways in stroke and possibly other neurodegenerative diseases associated with excitotoxicity.

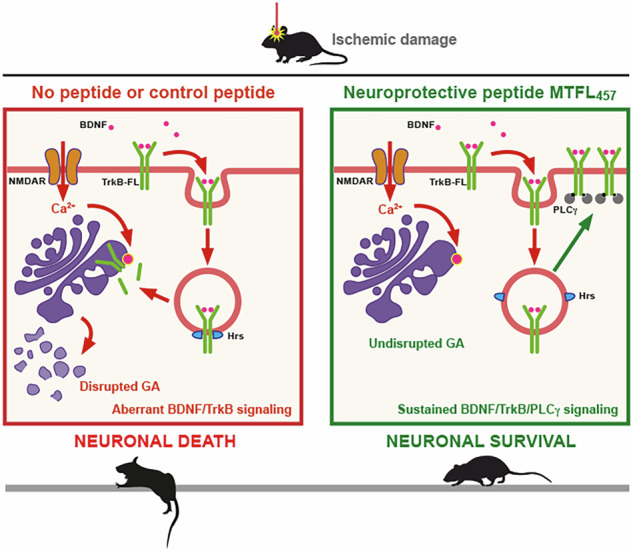

## Introduction

Stroke is a major global health concern, currently a leading cause of death, disability, and dementia, with its burden expected to increase due to population aging and a higher prevalence of vascular risk factors. However, effective therapies are lacking. For ischemic stroke, treatments primarily involve mechanical or pharmacological blood flow restoration, but these reach only a limited number of patients. Therefore, developing new, safe, and efficient neuroprotective treatments to reduce stroke’s impact is essential. Disruption of cerebral blood flow generates an irreversibly damaged infarct core surrounded by a potentially recoverable “penumbra” area. This region, however, can undergo secondary neuronal death, mainly driven by excitotoxicity resulting from overstimulation of N-methyl-D-aspartate glutamate receptors (NMDARs) [1], causing infarct expansion. Consequently, current neuroprotective strategies focus on slowing penumbra evolution by interfering with excitotoxicity. This process is also associated with many other CNS disorders, including hypoglycemia, epilepsy, acute trauma, and neurodegenerative diseases (NDDs) [2], reinforcing the value of excitotoxicity-targeted therapies. While NMDAR antagonists failed as neuroprotectants due to a lack of specificity, targeting NMDAR-downstream signaling has shown promise [3]. Clinical-stage results have been obtained for acute ischemic stroke [4, 5] using nerinetide, a peptide that uncouples NMDAR overactivation from neurotoxic signaling by dissociating the ternary complex of GluN2B NMDAR-subunits, postsynaptic density protein-95 (PSD-95), and neuronal nitric oxide synthase (nNOS) [6]. This approach utilizes cell-penetrating peptides (CPPs), promising molecules for CNS treatment that act as carriers to deliver therapeutic agents across the blood–brain barrier (BBB) and plasma membrane [7]. We have also employed CPPs to investigate complementary strategies aimed at preventing the aberrant excitotoxic downregulation of survival pathways, such as those regulated by the full-length tropomyosin-related kinase B receptor (TrkB-FL) [8] or PSD-95 [9].

Neuronal survival is promoted by brain-derived neurotrophic factor (BDNF) binding to its high-affinity receptor TrkB-FL, leading to increased tyrosine kinase (TK) activity and transphosphorylation of residues Y515 and Y816, anchor sites for adaptor proteins [10]. This activates interconnected pathways, including a cascade regulated by phospholipase C γ (PLCγ) [10, 11] that activates prosurvival transcription factors (TFs) such as cAMP response-element binding protein (CREB) [12] and myocyte enhancer factor 2 (MEF2) [13, 14]. Their targets include genes encoding TrkB [15, 16] and BDNF [17–19]. Upon neurotrophin binding, both TrkB-FL and TrkB-T1 (a truncated, TK-deficient isoform considered a dominant-negative neuronal receptor [20]) are rapidly internalized [21, 22], forming signaling endosomes [22]. Recycling to the cell surface is less efficient for TrkB-FL, depends on its TK activity, and is regulated by binding of the interdomain region separating transmembrane (TM) and TK sequences to the endosomal protein hepatocyte growth factor-regulated tyrosine kinase substrate (Hrs) [22].

TrkB signaling is profoundly aberrant in stroke and NDDs [23, 24], making receptor isoforms potential therapeutic targets for neuroprotection in excitotoxicity-associated diseases. Three mechanisms contribute to TrkB dysregulation: 1) transcriptional regulation favoring TrkB-T1 over TrkB-FL expression [25, 26]; 2) TrkB-FL cleavage by calpain producing a truncated receptor alike to TrkB-T1 [26] and a cytosolic 32-kDa fragment (f32) [25] presenting TK activity and nuclear accumulation [27]; and 3) regulated intramembrane proteolysis (RIP) of both isoforms by metalloproteinase (MP)/*γ*-secretase action, releasing identical BDNF-sequestering ectodomains [26]. In stroke, TrkB-FL regulation is primarily due to calpain processing, with RIP being secondary. We previously demonstrated that TrkB-FL is depleted from the neuronal surface early in excitotoxicity, and both calpain and RIP processing require prior endocytosis [8], suggesting intracellular cleavage. To preserve BDNF/TrkB-FL survival cascades during excitotoxicity, we developed MTFL_457_, an HIV Tat-derived CPP containing a TM/TK interdomain sequence that maintains TrkB-FL at the cell surface, away from activated proteolytic machinery, thereby preventing processing by calpain and MPs [8]. Consequently, neuronal viability was preserved via a PLC*γ*-dependent cascade that maintains downstream CREB and MEF2 promoter activities and increased expression of critical prosurvival proteins, initiating a feedback mechanism. This neuroprotective CPP may be relevant for human stroke therapy, as it also prevents TrkB-FL downregulation, reduces infarct size, and improves neurological outcome after experimental stroke in mice [8].

In this work, we demonstrate that TrkB-FL endocytosis induced by excitotoxicity is followed by interaction with Hrs and retrograde transport to the Golgi apparatus (GA), coinciding with organelle dispersion. Importantly, GA disruption is considered a relevant hallmark of neuronal damage which precedes neurodegeneration [28]. Interestingly, MTFL_457_ inhibits TrkB-FL/Hrs interaction and blocks TrkB-FL retrograde transport, protecting neurons from organelle fragmentation and excitotoxicity. Thus, our results unveil a central role for TrkB-FL in GA stability and suggest that the neuroprotective peptide MTFL_457_ could be relevant not only for treating stroke but also NDDs associated with excitotoxicity and GA disruption.

## Results

### MTFL_457_ preserves cell-surface pY816-TrkB-FL away from proteolytic machinery activated by excitotoxicity

**Fig. 1:**
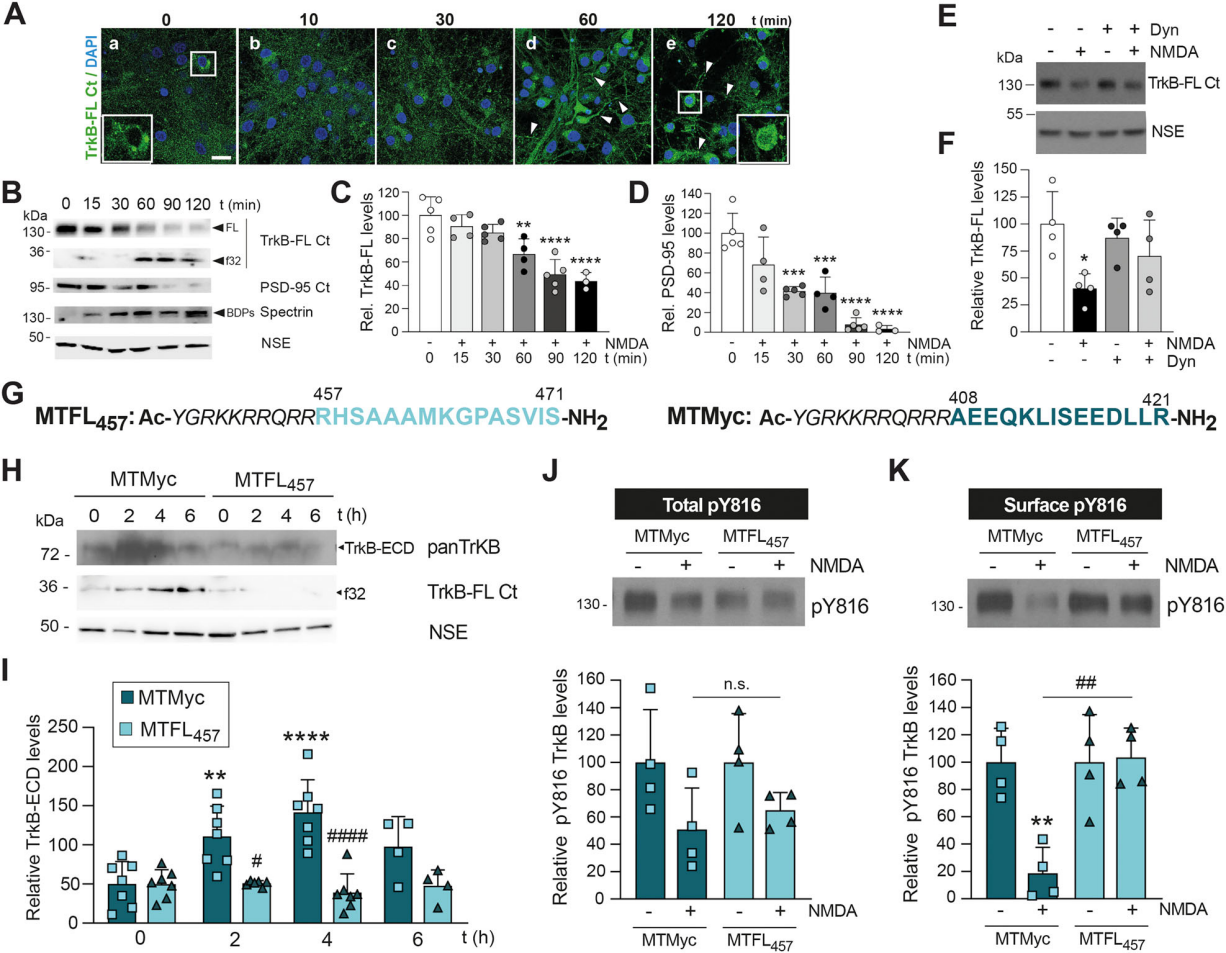
MTFL_457_ preserves pY816-TrkB-FL at the cell surface, protecting it from proteolytic machinery activated secondarily by excitotoxicity. **A**–**D** Kinetics of TrkB-FL downregulation. Primary cortical cultures were treated with 100 μM NMDA and its co-agonist 10 μM glycine (hereafter referred to as ‘NMDA’). Immunofluorescence (**A**) and immunoblotting (**B**) used a C-terminal (C-ter) isoform-specific antibody (TrkB-FL Ct) recognizing both the full-length protein (FL) and the intracellular fragment (f32). **A** Shows TrkB-FL (green) and nuclei (blue, DAPI stain). Arrowheads indicate varicosities in neuronal projections. Scale bar: 20 μm. Insets show cell body details for untreated cells and cells treated with NMDA for 120 min. **B** Compares the decrease in TrkB-FL and formation of f32 with PSD-95 downregulation, detected using a C-terminal antibody (PSD-95 Ct). Calpain activation was confirmed by the accumulation of characteristic spectrin breakdown products (BDPs; 150 and 145 kDa). Neuron-specific enolase (NSE) served as a loading control for protein normalization. **C**, **D** Quantification of normalized TrkB-FL and PSD-95 levels, shown relative to levels in the absence of NMDA (control). Data are represented as means ± SD. Statistical analysis: one-way analysis of variance (ANOVA) followed by Bonferroni *post hoc* test (**P < 0.01, ***P < 0.001, ****P < 0.0001; 0-90 min, *n* = 5; 120 min, *n* = 3). **E**, **F** Effect of dynasore preincubation (80 μM, 30 min) on TrkB-FL levels after 2 h NMDA treatment. Data are means ± SD (*n* = 4); statistical analysis as above (*P < 0.05). **G** Sequences of the cell-penetrating neuroprotective peptide (MTFL_457_) and control peptide (MTMyc). Both contain Tat amino acids (aa) 47–57 (italic), followed by rat TrkB-FL aa 457-471 (light blue) or c-Myc aa 408-421 (dark blue), respectively. **H**, **I** Effect of peptide preincubation (25 μM, 30 min) on TrkB-FL shedding and processing during NMDA treatment. Culture media were analyzed using an antibody against the TrkB-FL extracellular domain (panTrkB), which recognizes the ectodomains (ECDs) of all isoforms (TrkB-ECD), to assess receptor shedding by metalloproteinase (MP) activation. Total lysates were analyzed with the TrkB-FL Ct antibody to evaluate TrkB-FL calpain processing via f32 production. Relative TrkB-ECD levels are shown as means ± SD (0-4 h, *n* = 7; 6 h, *n* = 4). Statistical analysis: two-way ANOVA followed by Bonferroni *post hoc* test (**P < 0.01, ****P < 0.0001, comparing each peptide + NMDA vs. peptide alone; ^#^ *P* < 0.05, ^####^ *P* < 0.0001, comparing MTMyc vs. MTFL_457_ at each time point). **J**, **K** Effect of NMDA on total and cell-surface pY816-TrkB-FL levels. Cultures were preincubated with peptides as above, then treated briefly with NMDA (1 h) to minimize receptor degradation. Cell-surface proteins were biotin-labeled and precipitated, then compared to corresponding total lysates. Data are represented as means ± SD (*n* = 4). Statistical analysis: two-way ANOVA followed by Bonferroni *post hoc* test (*n.s*. = *n*ot significant; **P < 0.01, comparing NMDA vs. no NMDA; ^##^ *P* < 0.01, comparing MTMyc + NMDA vs. MTFL_457_ + NMDA).

Previous results suggested that processing was not the primary mechanism of TrkB-FL regulation in excitotoxicity [8]. Analysis of receptor downregulation at early times of in vitro excitotoxicity showed characteristic TrkB-FL labelling (Fig. 1A, panel a) maintained up to 30 min of damage (Fig. 1A, panels b, c). NMDA treatment then progressively decreased the TrkB-FL signal (Fig. 1A, panels d, e), particularly in dendrites presenting focal swellings and varicosities (arrowheads) characteristic of excitotoxicity preceding death [29]. For the longest treatment, partial nuclear accumulation was also observed (Fig. 1A, inset in panel e), probably due to f32 translocation [27]. Changes in TrkB-FL levels were not statistically significant until 60 min (Fig. 1B, C); this timing paralleled f32 increase and was concomitant with calpain activation, established by cleavage of its well-characterized substrate spectrin into breakdown products (BDPs, Fig. 1B). Downregulation of additional calpain substrates such as PSD-95 [9] and brain spectrin [30] was faster (Fig. 1B, D) and specific, as levels of neuronal specific enolase (NSE) were not significantly affected. Support for TrkB-FL processing being secondary also came from dynasore inhibition of dynamin-dependent endocytosis, which resulted in TrkB-FL stabilization during excitotoxicity (Fig. 1E, F), as previously demonstrated in ischemia [8]. Moreover, in contrast to the control peptide MTMyc (Fig. 1G), which had no effects on TrkB-FL downregulation or neuronal viability [8], MTFL_457_ prevented progressive f32 accumulation and NMDA-induced shedding of the highly glycosylated receptor ectodomain (TrkB-ECD, Fig. 1H, I). Interestingly, this TrkB-derived peptide lacks sequences presumably recognized by calpain and MPs, indicating it must affect receptor processing indirectly. In fact, MTFL_457_ prevented pY816-TrkB-FL endocytosis and preserved this partially active receptor at the cell surface (Fig. 1J, K). Conversely, a brief NMDA treatment strongly induced receptor endocytosis in MTMyc-treated cultures, likely due to the general triggering of endocytic processes by excitotoxicity [31, 32].

### MTFL_457_ provides long-term neuroprotection in excitotoxicity and ischemia, partially dependent on the TrkB-FL KFG domain

**Fig. 2:**
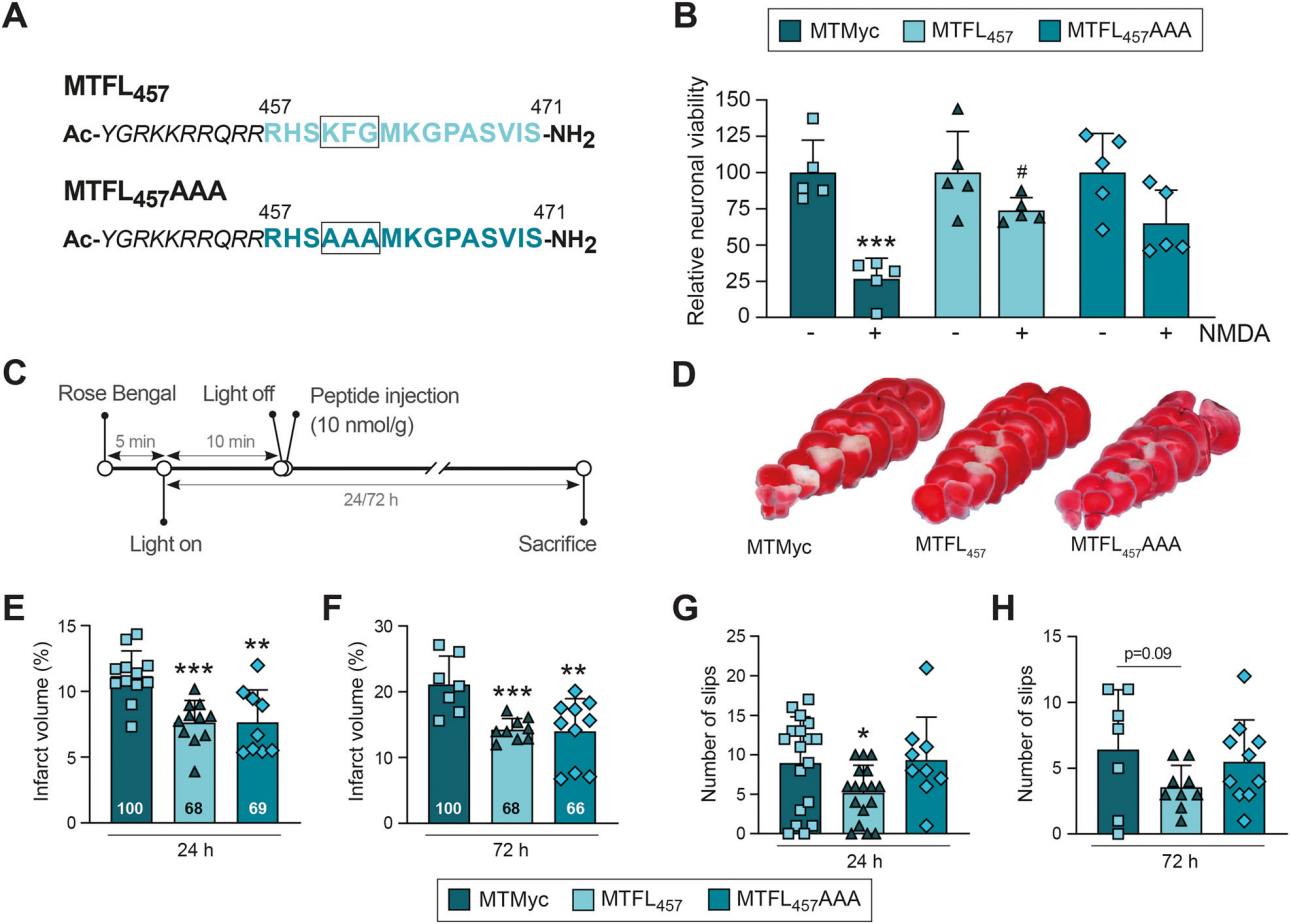
MTFL_457_ has long-term neuroprotective effects in excitotoxicity and ischemia which are partially dependent on the TrkB-FL KFG domain. **A** Comparison of MTFL_457_ and MTFL_457_AAA sequences. The latter contains Tat aa 47–57 (italic) followed by the indicated rat TrkB-FL sequence but substituting the highly conserved KFG domain with AAA. **B** Effect of MTFL_457_AAA on neuronal viability in cultures preincubated with MTMyc, MTFL_457_ or MTFL_457_AAA as before, and treated with NMDA (2 h). For each peptide, results obtained in excitotoxic conditions were expressed relative to those obtained in the absence of NMDA. Five completely independent experiments were carried out, each including sample triplicates for every condition tested. Means ± SD are represented, and statistical analysis was performed by two-way ANOVA followed by a Bonferroni test (***P < 0.001 for NMDA effect on MTMyc-treated cells; ^#^ *P* < 0.05 for MTFL_457_ vs. MTMyc effect on NMDA-treated cells). **C** Timeline to analyze in vivo effects of MTMyc, MTFL_457_ or MTFL_457_AAA in the model of ischemia. Microvascular photothrombotic permanent damage was initiated in mice by cold-light irradiation (10 min) of a stereotaxically selected brain area after *i.v*. injection of photosensitive dye Rose Bengal as detailed in Materials and methods. CPPs (10 nmol/g) were retro-orbitally administered 10 min after damage initiation, and animals were sacrificed 24 h or 72 h later. **D** Representative 1 mm brain coronal slices stained with TTC corresponding to animals injected with MTMyc, MTFL_457_ or MTFL_457_AAA and sacrificed at 24 h. E and F Infarct volume of CPP-injected animals expressed as a percentage of the contralateral hemisphere volume. Means ± SD are given for animals sacrificed 24 h (**E**; *n* = 9-12) or 72 h (**F**; *n* = 7-10) after damage initiation. Infarct volumes for MTFL_457_ and MTFL_457_AAA experimental groups are also expressed as a percentage of values obtained in animals injected with MTMyc. **G**, **H** Evaluation of balance and motor coordination. The number of contralateral hind paw slips was measured in animals 24 h (**G**, *n* = 9-17) or 72 h (**H**, *n* = 7-10) after damage induction. **E**–**H** Differences with control animals (MTMyc group) were analyzed by Student’s *t*-test (*P < 0.05, **P < 0.01, ***P < 0.001).

How TrkB-FL endocytosis is regulated in pathological contexts like excitotoxicity is not well characterized. Although controversial regarding TrkB receptors [33], BDNF-induced Trk endocytosis is controlled by ubiquitination of residue K460 [34], located inside a highly conserved KFG domain. MTFL_457_ contains this and surrounding residues; therefore, it might potentially prevent NMDA-induced TrkB ubiquitination and endocytosis. In contrast, a peptide lacking the KFG sequence (MTFL_457_AAA, Fig. 2A) might not affect neuronal viability in vitro or in vivo. However, MTFL_457_ and MTFL_457_AAA similarly prevented the significant NMDA-induced decrease in neuronal viability in MTMyc-preincubated cultures, although differences did not reach statistical significance for MTFL_457_AAA (Fig. 2B). For in vivo experiments, we used a microvascular photothrombosis model (Fig. 2C) [35]. This produced permanent vessel occlusion and focal cortical infarcts in motor and somatosensory areas of the ipsilateral hemisphere, visualized as TTC-unstained regions (Fig. 2D). Infarct volumes in animals treated with MTFL_457_ or MTFL_457_AAA 10 min after injury were very similar and significantly lower than values obtained with MTMyc, both at 24 h (Fig. 2E) and 72 h (Fig. 2F) of damage, representing reductions higher than 31%. Treatment 1 h after injury only showed a trend towards reduced infarct volumes (Fig. S1). Interestingly, the decrease in infarct volume induced by MTFL_457_ treatment (10 min post-injury) correlated with improved balance and motor coordination in the beam walking test, which was significant at 24 h (Fig. 2G) but not 72 h (Fig. 2H; *P* = 0.09). Conversely, this correlation was not observed in animals treated with MTFL_457_AAA at either time point. Therefore, although the KFG sequence is not required for MTFL_457_ ability to interfere with in vitro and in vivo excitotoxicity, it might be involved in additional protective functions exerted by TrkB-FL in neurons or alternative neural cells.

### Effect of excitotoxicity on TrkB-FL interaction with endosomal protein Hrs and MTFL_457_ regulation

**Fig. 3:**
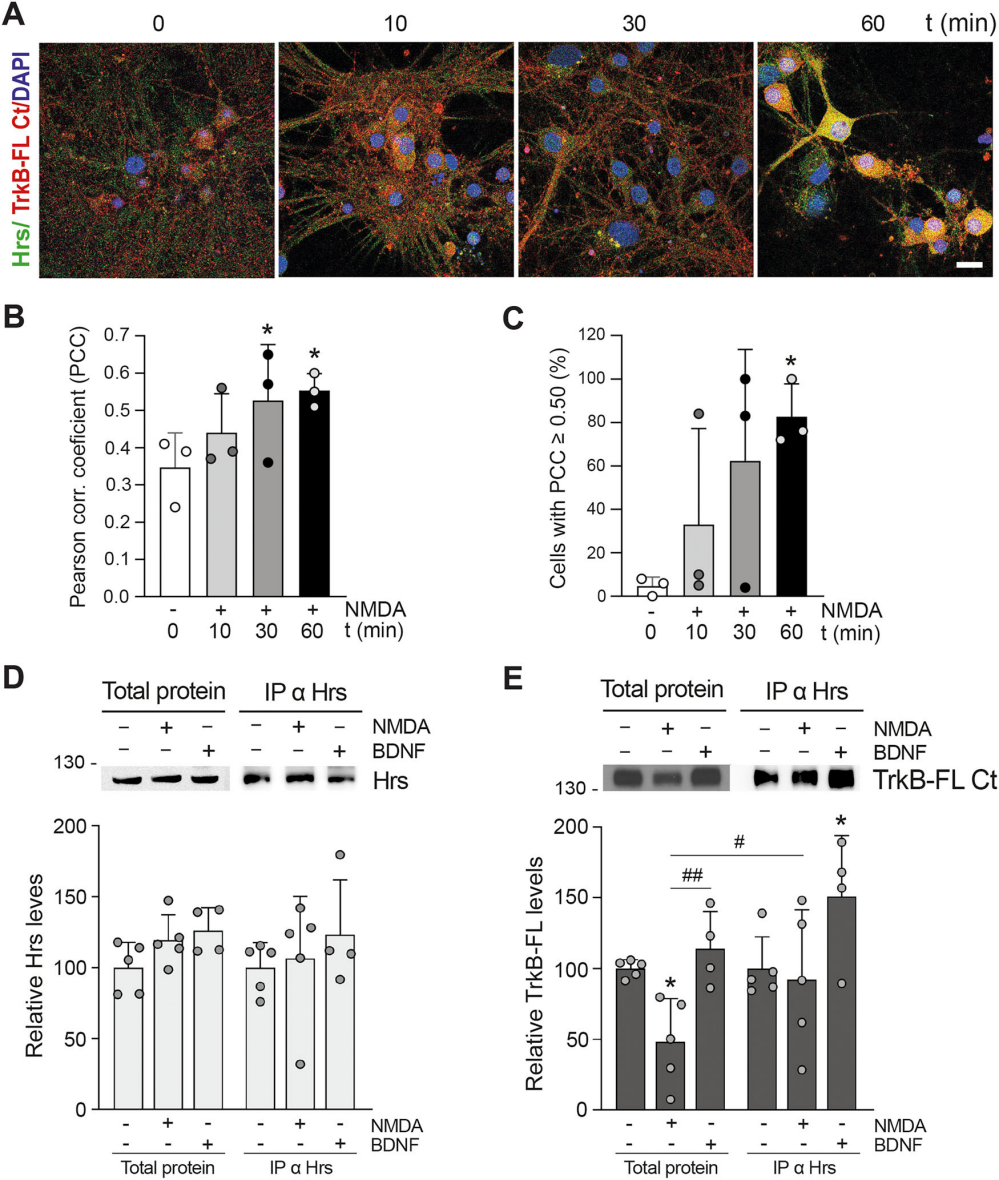
Effect of excitotoxicity on TrkB-FL interaction with endosomal protein Hrs. **A**–**C** Analysis by immunofluorescence of TrkB-FL/Hrs colocalization. **A** Cortical neurons were treated with NMDA for the indicated times and analyzed with antibodies specific for Hrs (green) and TrkB-FL Ct (red); nuclear staining was performed with DAPI (blue). Representative images obtained by confocal microscopy correspond to single sections and show the fused channels. Scale bar: 20 μm. **B** Mean values ± SD of Pearson correlation coefficient (PCC; *n* = 3). For each independent experiment, a minimum of 80 different neurons were analyzed. Statistical analysis was performed using a generalized linear model followed by a *post-hoc* Fisher’s LSD test (*P < 0.05, compared to untreated cultures). **C** Percentage of cells showing at different times of NMDA treatment a PCC ≥ 0.50, generally considered as a threshold for protein colocalization. Statistical analysis was performed as before (* P < 0.05, compared to untreated cultures; *n* = 3). **D**, **E** Analysis by immunoprecipitation of TrkB-FL/Hrs interaction. Neuronal cultures were treated with NMDA (100 μM) or BDNF (100 ng/ml) for 30 min and compared to untreated cultures. Immunoprecipitation was performed with the Hrs antibody, and the immunoprecipitated proteins (IP) were analyzed by immunoblot using the same antibody (**D**) or TrkB-FL Ct (**E**). Total protein lysates were analyzed in parallel with the immunoprecipitated proteins. Mean values ± SD (*n* = 5, except for BDNF-treated cells where *n* = 4) of Hrs and TrkB-FL levels in NMDA- or BDNF-treated cultures relative to untreated cells are represented for both total lysates and Hrs-immunoprecipitated proteins. Statistical analysis was performed as above (*P < 0.05, compared to the respective untreated cultures; ^#^*P* < 0.05 and ^##^*P* < 0.01, as indicated).

Cell-surface membrane protein abundance depends on integrating secretory and endolysosomal pathways, establishing a steady-state proteome actively remodeled by physiologic needs. Disrupting this balance is associated with pathological conditions, particularly aging-related diseases and neurodegeneration [36]. In the BDNF response, the TrkB-FL juxtamembrane intracellular region L453-N536 interacts with Hrs, an early and late endosomal protein [37] regulating the balance between partial receptor lysosomal degradation or recycling to the membrane [22, 37, 38]. We hypothesized that excitotoxicity might profoundly alter this Hrs-regulated balance. To test this hypothesis, we first investigated the effect of excitotoxicity on Hrs levels, finding no changes after brief (Fig. S2A) or prolonged (Fig. S2B, C) induction. Next, we analyzed TrkB-FL/Hrs interaction under basal versus excitotoxic conditions. Colocalization was low basally (Fig. 3A), corresponding to a Pearson correlation coefficient (PCC) below a 0.50 threshold value (Fig. 3B). Excitotoxicity induced a progressive increase in colocalization (Fig. 3A), the PCC reaching 0.56 after 60 min (Fig. 3B), when 83 ± 15% of neurons showed a PCC ≥ 0.50 (Fig. 3C). Coimmunoprecipitation with Hrs antibodies (Fig. 3D, E) or a generic TrkB antibody (panTrkB; Fig. S3) confirmed these findings. In cultures briefly treated with NMDA or BDNF (internal control), we confirmed stable Hrs levels in total extracts and comparable immunoprecipitation by the Hrs antibody (Fig. 3D). In contrast, although NMDA significantly decreased total TrkB-FL, similar levels coimmunoprecipitated with the Hrs antibody (Fig. 3E). The difference between relative TrkB-FL levels in total extracts versus those Hrs-coimmunoprecipitated was statistically significant during excitotoxicity. As described, BDNF treatment increased TrkB-FL/Hrs interaction [22]. Using panTrkB, we confirmed TrkB-FL precipitation with the isoform-specific antibody TrkB-FL Ct (Fig. S3A), finding reduced receptor levels in NMDA-treated cells relative to those in BDNF presence, corresponding to total TrkB-FL levels. However, no parallel decrease was observed in coimmunoprecipitated Hrs (Fig. S3B).

**Fig. 4:**
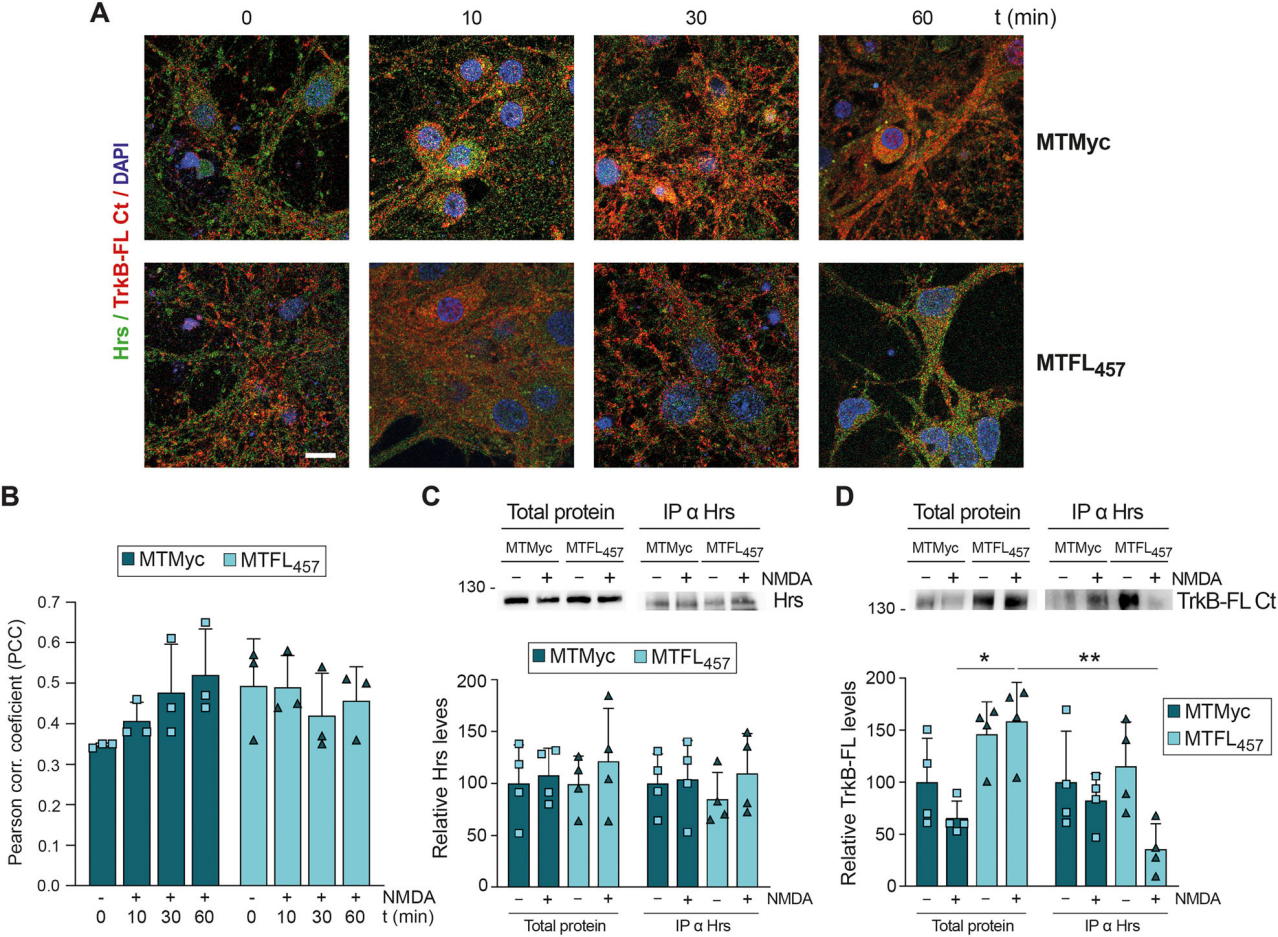
Regulation by MTFL_457_ of the TrkB-FL/Hrs interaction induced by excitotoxicity. **A** Cortical neurons were preincubated with MTMyc and MTFL_457_ (25 μM, 30 min) and treated with NMDA for the indicated times. Cells were analyzed by immunofluorescence with antibodies for Hrs (green) and TrkB-FL Ct (red), together with DAPI staining (blue). Representative confocal microscopy images correspond to single sections and show the fused channels. Scale bar: 10 μm. **B** Mean values ± SD of Pearson correlation coefficient (PCC; *n* = 3). For each independent experiment, a minimum of 80 different neurons were analyzed. Statistical analysis was performed using a generalized linear model followed by a *post-hoc* Fisher’s LSD test (*P* = 0.06, 0 vs. 60 min of NMDA treatment in MTMyc-cultures). **C**, **D** Analysis by immunoprecipitation of TrkB-FL/Hrs interaction. Cultures preincubated with cell-penetrating peptides (CPPs) as above were treated with NMDA for 30 min and compared to untreated cultures. Immunoprecipitation (IP) was performed with the Hrs antibody, and the immunoprecipitated proteins were analyzed by immunoblot using the same antibody (**C**) or TrkB-FL Ct (**D**). Total protein lysates and immunoprecipitated proteins were analyzed in parallel. Mean values ± SD (*n* = 4) of Hrs and TrkB-FL levels relative to those found in cells preincubated with MTMyc and without NMDA are represented. Statistical analysis was performed using two-way ANOVA followed by a Bonferroni test (*P < 0.05, **P < 0.01).

MTFL_457_ contains TrkB-FL residues inner to the region involved in Hrs interaction. Therefore, we characterized a possible peptide effect on TrkB-FL/Hrs colocalization (Fig. 4). In MTMyc-cultures, excitotoxicity showed a tendency toward increased colocalization (Fig. 4A, B), similarly to results obtained without peptide. In contrast, colocalization was unaffected by excitotoxicity in the presence of MTFL_457_. Coimmunoprecipitation experiments confirmed immunoprecipitation of equivalent Hrs amounts (Fig. 4C) while TrkB-FL/Hrs interaction was favored by excitotoxicity in MTMyc-cultures (Fig. 4D). Conversely, although TrkB-FL was stabilized by MTFL_457_ action, a significant decrease in TrkB-FL/Hrs interaction was observed under excitotoxic conditions in MTFL_457_-cultures. We conclude that from early times of NMDAR overactivation, Hrs interaction is promoted and regulates TrkB-FL fate. Disruption by MTFL_457_ of TrkB-FL/Hrs interaction might secondarily prevent excitotoxicity-induced TrkB-FL proteolysis, resulting in neuroprotection.

### Effect of excitotoxicity on TrkB-FL transport to the GA and MTFL_457_ regulation

**Fig. 5:**
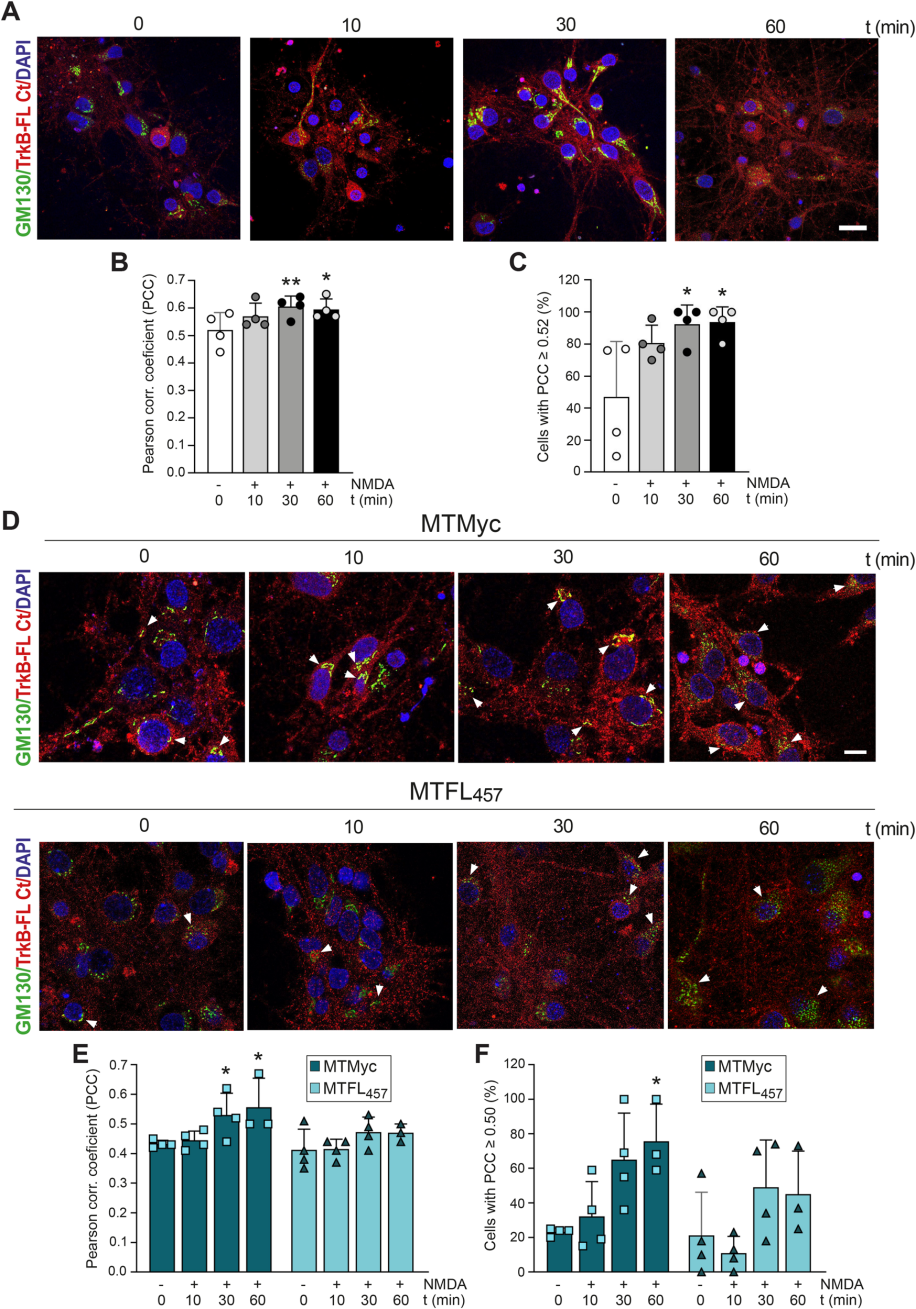
Effect of excitotoxicity on TrkB-FL transport to the Golgi complex and regulation by MTFL_457_ action. Cortical neurons were treated with NMDA for the indicated times and analyzed by immunofluorescence with antibodies specific for the Golgi matrix protein GM130 (green) and TrkB-FL Ct (red); nuclear staining was performed with DAPI (blue). Excitotoxicity was induced in the absence of peptides (**A**–**C**) or after preincubation with MTMyc and MTFL_457_ (25 μM, 30 min) (**D**–**F**). **A**, **D** Representative images obtained by confocal microscopy corresponding to single sections, showing channels fused. Scale bar: 20 μm. **B**, **E** Mean PCC values ± SD (0-30 min, *n* = 4; 60 min, *n* = 3) for TrkB-FL and GM130 colocalization. Statistical analysis was performed using a generalized linear model followed by a *post-hoc* Fisher’s LSD test (*P < 0.05, **P < 0.01, compared to basal conditions without peptide or with MTMyc, respectively). **C**, **F** Percentage of cells showing, at different treatment times, a PCC ≥ 0.52 (**C**), the value obtained for TrkB-FL/GM130 colocalization in basal conditions in the absence of peptide, or ≥ 0.50 (**F**). Statistical analysis was performed by one-way ANOVA followed by a Bonferroni *post hoc* test (*P < 0.05, compared to basal conditions without peptide or with MTMyc, respectively; 0–30 min, *n* = 4; 60 min, *n* = 3). For each independent experiment, a minimum of 80 different neurons were analyzed.

Retrograde GA protein transport allows some cargoes to avoid lysosomal degradation, although they remain susceptible to proteolysis within that organelle. For instance, the TrkB/NMDAR shared effector Kidins220 [39] undergoes early endocytosis, GA transport, and subsequent calpain-dependent degradation following excitotoxicity [40]. Accordingly, we characterized TrkB-FL GA trafficking after brief NMDA treatment using immunofluorescence with antibodies against TrkB-FL Ct and GM130, a Golgi matrix protein central to organelle preservation, protein glycosylation, and vesicle transport. In basal conditions, GM130 showed a characteristic GA distribution in front of apical dendrites and appeared as flat cisternae (Fig. 5A), coinciding with strong TrkB-FL presence (Fig. 5B), as described [41]. NMDA treatment further increased TrkB-FL/GM130 colocalization (Fig. 5B), occurring in most neurons after 30 min (Fig. 5C). Similar results were obtained in cultures preincubated with MTMyc (Fig. 5D-F), while TrkB-FL distribution was maintained in neurons preincubated with MTFL_457_. These findings suggest that early in excitotoxicity, a TrkB-FL fraction is recruited towards the GA before processing, and peptide MTFL_457_ can prevent this recruitment.

### Effect of in vitro excitotoxicity on GA stability and MTFL_457_ regulation

**Fig. 6:**
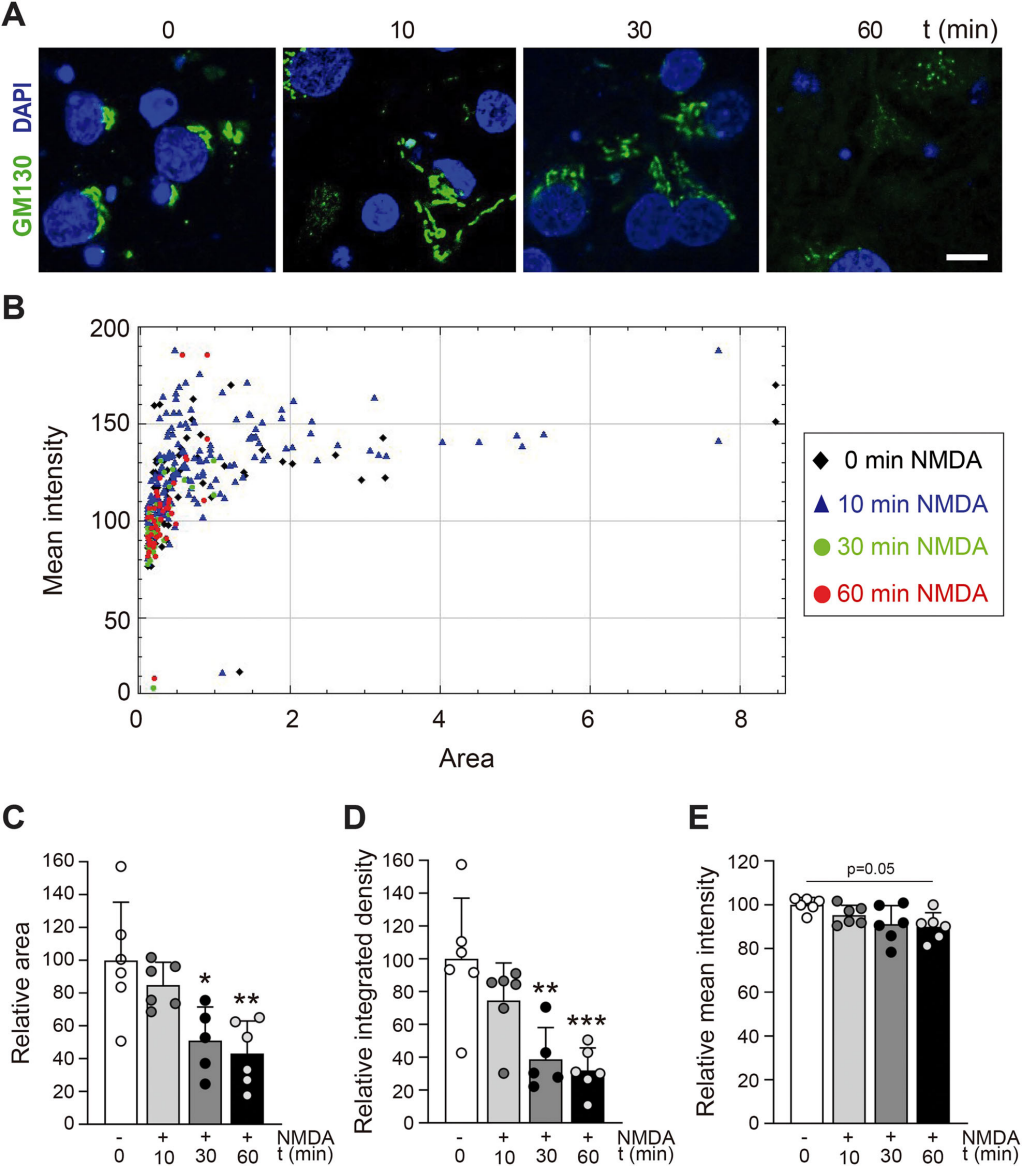
Effect of in vitro excitotoxicity on GA stability. Cortical neurons were treated with NMDA for the indicated times and analyzed by immunofluorescence with a GA-specific antibody (GM130, green), together with DAPI nuclear staining (blue). **A** Representative images obtained by confocal microscopy corresponding to single sections. Scale bar: 10 μm. **B** Representation of area versus mean fluorescence intensity for each GA particle detected in representative images from different times of NMDA treatment. **C**–**E** Mean values ± SD (*n* = 6) of particle area (**C**), integrated density (**D**) and mean fluorescence intensity (**E**). An average of 80 particles were quantified per condition in each independent experiment analyzed. In all cases, statistical analysis was performed using one-way ANOVA followed by the Bonferroni test (*P < 0.05, **P < 0.01, ***P < 0.001, compared to untreated cultures).

**Fig. 7:**
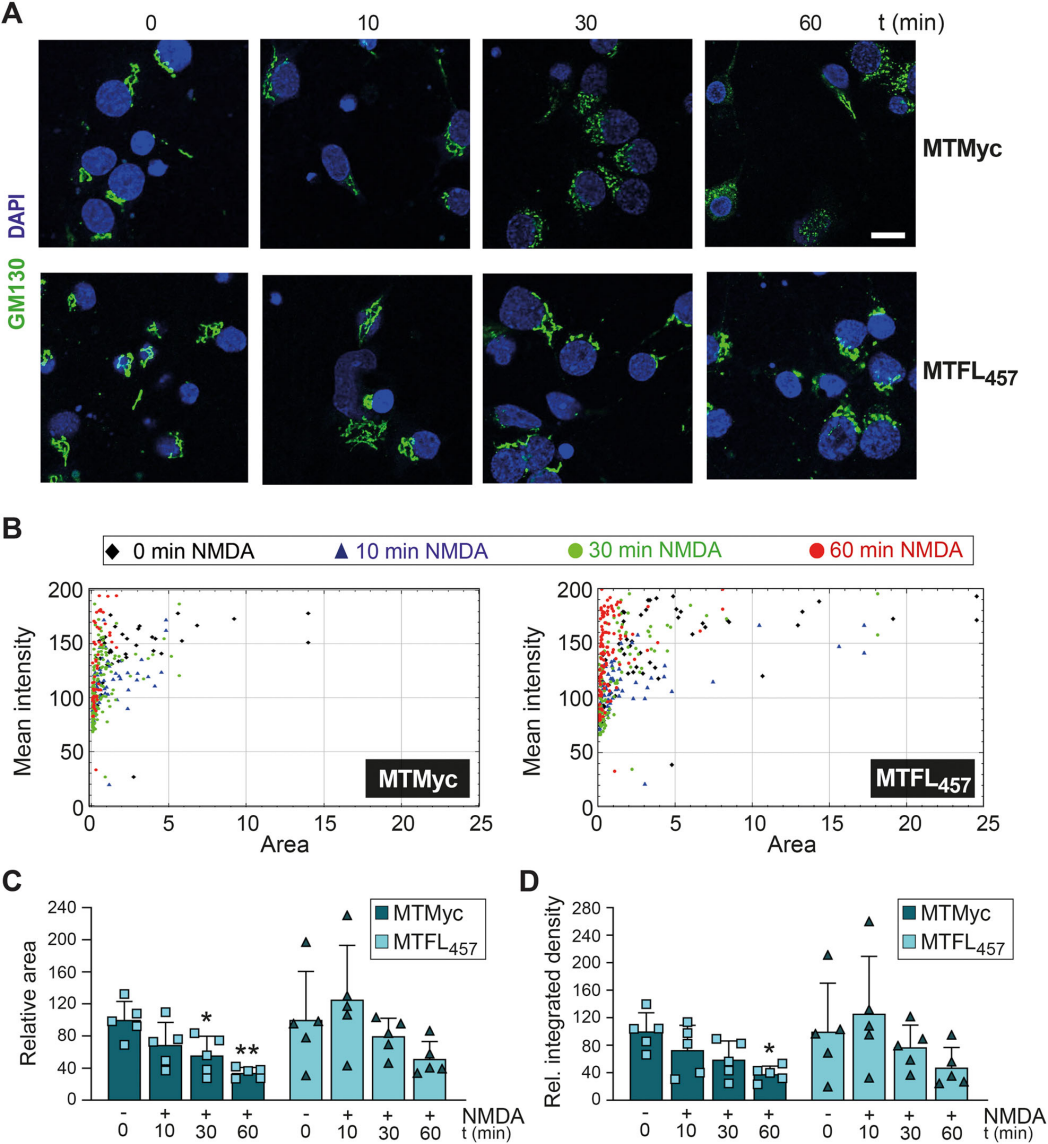
Regulation of excitotoxicity-induced GA disruption by peptide MTFL_457_. Cortical neurons preincubated with MTMyc and MTFL_457_ (25 μM, 30 min) and treated with NMDA for the indicated times were analyzed by immunofluorescence with a GA-specific antibody (GM130, green) and DAPI (blue). **A** Representative images obtained by confocal microscopy corresponding to single sections. Scale bar: 10 μm. **B** Representation of the area versus the mean fluorescence intensity for each GA particle detected in representative images corresponding to cultures preincubated with MTMyc (left panel) or MTFL_457_ (right panel), treated with NMDA for the indicated times. **C**, **D** Mean values ± SD (*n* = 5) of the particle area (**C**) and integrated density (**D**). Statistical analysis was performed using one-way ANOVA followed by the Bonferroni test (*P < 0.05, **P < 0.05, compared to basal conditions with MTMyc). An average of 80 particles were quantified per condition in each independent experiment analyzed.

Golgi fragmentation and dispersal precede neuronal death from excitotoxicity and other insults [42]. We therefore analyzed GA stability during early in vitro excitotoxicity and how it might be affected by the neuroprotective peptide MTFL_457_. Unlike the condensed, flattened cisternae in untreated neurons, excitotoxicity induced significant GA fragmentation into scattered particles (Fig. 6A). A representative experiment showed gradual, parallel decreases in the mean fluorescence intensity and area of detected structures with treatment time (Fig. 6B). Resulting differences in area (Fig. 6C) and integrated density (Fig. 6D) were statistically significant due to GA fragmentation, with trends towards decreased mean intensity (Fig. 6E) and increased particle circularity (data not shown). These results confirmed GA disruption by excitotoxicity, occurring with kinetics similar to TrkB-FL retrograde transport. Accumulation of TrkB-FL via excitotoxicity-induced retrograde transport might promote GA fragmentation; therefore, MTFL_457_ prevention of this traffic might also protect neurons from organelle disruption. Indeed, GA fragmentation was remarkably decreased after MTFL_457_ pre-treatment compared to MTMyc (Fig. 7A). In a representative experiment, MTFL_457_-treated neurons showed higher particle intensity and area values than MTMyc-preincubated neurons (Fig. 7B). Accordingly, NMDA induced increased circularity (mainly associated with small particles) in MTMyc-treated neurons (Fig. S4, left panel), while MTFL_457_-treated cells showed a more dispersed distribution (Fig. S4, right panel). Significant excitotoxicity-induced differences in particle area (Fig. 7C) and integrated density (Fig. 7D) in MTMyc cultures were lost with MTFL_457_. These results demonstrate that GA fragmentation is an early excitotoxic event preceding nuclear changes, involving loss of the condensed GA structure in favor of smaller, more rounded particles, possibly vesicular fragments. MTFL_457_, a neuroprotective peptide preventing TrkB-FL retrograde transport and degradation in excitotoxicity, also partially maintains GA integrity. Therefore, TrkB-FL might play a role in regulating Golgi disruption during neurodegeneration.

### Effect of brain ischemia on GA stability and MTFL_457_ regulation

**Fig. 8:**
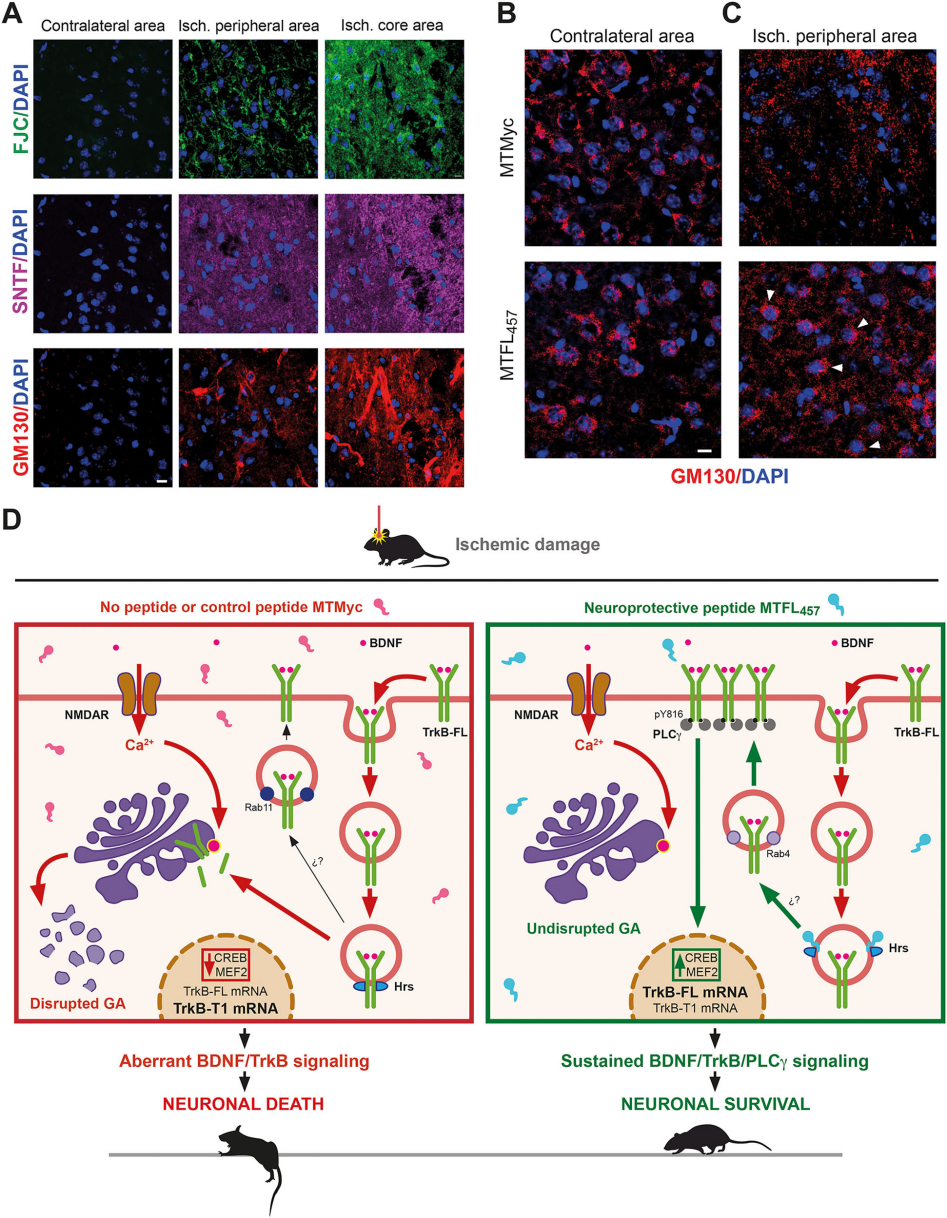
Effect of brain ischemia on GA stability, MTFL_457_ regulation, and proposed model. **A** Strong association between neuronal degeneration and serum protein leakage after ischemic damage. Immunohistochemistry of brain coronal sections from animals sacrificed 5 h after insult was performed with an antibody recognizing a calpain-generated neoepitope in spectrin N-terminal fragment (SNTF, magenta), labeling cells where this protease is overactive, and a mouse antibody recognizing GM130 (red). Neurodegeneration was also detected by Fluoro-Jade C (FJC) staining (green). Three different tissue areas were compared: the ischemic core, an area peripheral to the infarct core, and the equivalent area of the contralateral hemisphere. Leakage of mouse immunoglobulins due to early blood-brain barrier (BBB) breakage after the ischemic insult, detected by the secondary anti-mouse antibody (see Fig. S5), was observed in the neurodegenerating tissue and strongly interfered with GM130 detection. **B**, **C** GM130 staining after preincubation of coronal sections with an anti-mouse IgG (Fab specific) antibody to improve detection. Animals were retro-orbitally injected with peptides MTMyc or MTFL_457_ (10 nmol/g) 10 min after damage initiation and sacrificed 5 h later. Comparison of the contralateral (**B**) and the ischemic peripheral areas (**C**). Representative images correspond to single sections. Scale bar: 10 µm. **D** Model of TrkB-FL regulation in excitotoxicity and MTFL_457_ action. Endocytosis of neurotrophin receptor TrkB-FL is promoted by excitotoxicity in neurons treated with control peptide MTMyc or without treatment (left panel). In endosomes, TrkB-FL interacts with the protein Hrs and is retrogradely transported to the Golgi apparatus (GA), where activation of organelle-associated proteinases would be responsible for receptor processing by calpain and regulated intramembrane proteolysis (RIP). Although partial recycling back to the membrane might occur via mechanisms similar to those found after BDNF activation, there is a strong decrease in BDNF/TrkB-FL signaling and CREB/MEF2 promoter activities, causing transcriptional changes that favor neuronal death. In parallel, the GA is disrupted, a hallmark common to many neurodegenerative diseases (NDDs). The neuroprotective peptide MTFL_457_ interferes with the TrkB-FL/Hrs interaction induced by excitotoxicity, receptor retrograde transport and processing, as well as GA fragmentation (right panel). We propose that interference by MTFL_457_ with the TrkB-FL/Hrs interaction might favor rapid recycling back to the membrane, similar to that of isoform TrkB-T1, sustained BDNF/TrkB-FL/PLCγ signaling, and promotion of neuronal survival.

MTFL_457_ is efficiently delivered *i.v*. to the brain cortex and, in a permanent ischemia model [35, 43], affects TrkB-FL downregulation, infarct size, and neurological damage [8]. Whether GA disruption occurs in this model, which features a relatively narrow ischemic penumbra [44], or is affected by MTFL_457_, was unknown. In a transient ischemia model, damage to the GA was observed in penumbral neurons via undefined mechanisms [45]. We analyzed infarcts 5 h after photothrombosis (before stabilization [26]), comparing neurodegeneration across contralateral hemisphere, infarct core, and peripheral areas. Degenerating neurons were identified using Fluoro-Jade C (FJC) or immunohistochemistry with SNTF antibody, labeling cells with overactivated calpain (Fig. 8A). We observed no neurodegeneration contralaterally, high levels in the ischemic core (FJC/SNTF staining), and intermediate levels peripherally. Analysis of the GA using a mouse GM130 antibody was hampered by early BBB breakage after ischemia [46] and leakage of serum immunoglobulins into the ischemic brain. This caused high background and heavy blood vessel staining by secondary anti-mouse antibodies in the core (Fig. S5). The difficulty in using consistent visualization conditions necessitated internal comparisons between contralateral (Fig. S6A) and ischemic peripheral areas (Fig. S6B). In non-ischemic conditions, we observed a GA distribution similar to that in cultured neurons, with no peptide effect (Fig. S6A). Interestingly, in the peripheral ischemic tissue of MTFL_457_-treated animals, we observed GA staining resembling the contralateral region, along with lower backgrounds (Fig. S6B), likely from reduced BBB damage. Finally, coronal sections were preincubated with an anti-mouse IgG Fab fragment before GM130 detection to improve GA visualization (Fig. 8B, C). We observed a fragmented, dispersed GA in the peripheral ischemic tissue of MTMyc-treated animals, in contrast to those injected with MTFL_457_ (Fig. 8C), which again showed a GA distribution resembling the contralateral region (Fig. 8B). These results suggest TrkB-FL retrograde transport and processing might contribute to regulating stroke-associated GA fragmentation, and, consequently, strategies blocking receptor traffic and promoting GA stability could improve organelle function and contribute to neuronal survival after an ischemic insult.

## Discussion

We demonstrate here that the neurotrophin receptor TrkB-FL plays a key role in GA fragmentation during stroke neurodegeneration, a finding that encourages developing innovative strategies for neuroprotection in ischemia and other NDDs associated with excitotoxicity [2] and GA disruption [28]. Excitotoxicity causes rapid loss of cell-surface TrkB-FL and its recruitment to the GA, processes that precede and are required for organelle fragmentation (Fig. 8D, left panel). The GA is highly dynamic and essential for functions including trafficking/sorting of membrane and secretory proteins, and glycosylation of lipids and proteins. GA structure is modified by various physiological and pathological conditions. In mitotic cells, reversible organelle disassembly is required for mitosis entry [47]. Conversely, in post-mitotic neurons, the GA acts as a sensor of stress signals [42], and fragmentation precedes death induced by, among others, increased neuronal activity [42, 48]. Accordingly, GA disruption is an early event preceding further degeneration in stroke [45] and excitotoxicity-associated NDDs [2], including Alzheimer [49], Parkinson [50], Huntington [51], or amyotrophic lateral sclerosis [52]. However, the underlying neuronal mechanisms of GA damage remain poorly described. Interestingly, Golgi fragmentation is also induced by accumulation of autoactivated TrkB-FL (derived from heterologous overexpression or endogenous receptor transactivation) in the ER–Golgi intermediate compartment [53]. Therefore, increased TrkB-FL presence in the GA might regulate damage-associated organelle fragmentation. Recently, condensates formed by RNA and the GA-resident protein GM130 were identified as critical for Golgi ribbon integrity [54]. Future work should address whether TrkB-FL retrograde transport/degradation relates to this RNA/GM130 dissociation.

TrkB-FL intracellular trafficking under pathological conditions is not well characterized. Excitotoxicity rapidly decreases cell-surface receptors via endocytosis, similar to GluN2B-NMDARs [55], or Kidins220 and GluN1-NMDAR subunits, which are retrogradely transported to the GA [40]. Likewise, internalized TrkB-FL is transported to the GA during excitotoxicity (Fig. 8D, left panel), adding to the basal receptor pool there; this might constitute a stress signal promoting GA disruption and cell death. This retrograde transport requires Hrs interaction, suggesting Hrs regulates receptor trafficking after both BDNF [38] and NMDA stimulation. While Hrs normally balances TrkB-FL Rab11-dependent slow recycling and lysosomal degradation under physiological conditions, Hrs binding during excitotoxicity appears to favor retrograde transport over lysosomal degradation. Following endocytosis and retrograde transport, TrkB-FL is processed by MPs/*γ*-secretases and primarily calpain [26], a key protease in stroke and other excitotoxicity-related pathologies [30]. In addition to TrkB-FL [25], calpain also cleaves critical NMDAR-complex components critical to neuronal survival and function: scaffolding protein PSD-95 [9, 56], GluN2-NMDAR subunits [56], or Kidins220 [39, 57], similarly processed after GA transport [40]. Protease activation can occur near plasma and endosomal membranes [58], in high-[Ca^2+^] microdomains, or at subcellular compartments like the GA membrane [59, 60], nucleus, or mitochondria [61]. Excitotoxicity could activate GA-associated proteases, inducing proteolysis of cargoes like TrkB-FL within the GA (Fig. 8D, left panel). Altogether, these data unveil excitotoxicity-induced endocytosis, retrograde transport, and processing of prosurvival proteins as relevant neuroprotection targets [62].

The brain-accessible peptide MTFL_457_ was critical for establishing the importance of TrkB-FL retrograde transport for GA disruption and neurodegeneration in excitotoxicity. In addition to TrkB-FL endocytosis, MTFL_457_ prevents Hrs interaction, retrograde transport, organelle fragmentation, and TrkB-FL proteolysis (Fig. 8D, right panel), emphasizing the importance of residues 457-471 in controlling excitotoxicity-induced receptor trafficking. Further investigation could explore whether interfering with TrkB-FL/Hrs interaction promotes TrkB-FL Rab4-dependent fast recycling, similar to TrkB-T1 [22]. Interestingly, while the highly conserved KFG sequence present in MTFL_457_ is not required to interfere with in vitro and in vivo excitotoxicity, it might mediate other cytoprotective functions meriting investigation. MTFL_457_ action preserves a partially active pY816-TrkB-FL at the cell surface, supporting neuronal viability via a PLC*γ*-dependent mechanism likely via maintenance of CREB and MEF2 activities (Fig. 8D, right panel). These TFs start a feedback mechanism favoring expression of critical prosurvival proteins in excitotoxicity [8]. These results resemble those obtained with classical and fast-acting antidepressants (ADs), which directly bind TrkB-FL dimers and mediate their effects on neuroplasticity by stabilizing a receptor conformation that facilitates membrane retention, synaptic location, and BDNF accessibility [63]. Moreover, AD action depends on Y816-TrkB-FL phosphorylation and PLC*γ* signaling, and results in increased CREB phosphorylation, which mediates neuronal plasticity [64].

Neuroprotective peptide MTFL_457_ could be important for stroke treatment in humans because, in a preclinical stroke model, it not only counteracts TrkB-FL downregulation, decreases infarct size, and improves neurological outcome [8] but also inhibits GA fragmentation. However, before clinical translation, we need to address some study limitations, such as the influence of gender, age or stroke comorbidities in MTFL_457_ neuroprotection. In accordance to the Stroke Therapy Academic Industry Roundtable (STAIR) recommendations [65, 66], it would be also relevant to investigate if, in addition to the acute and early subacute stroke stages, MTFL_457_ neuroprotection lasts for post-stroke periods longer than 72 h. Regarding the optimal timing of treatment, although a tendency towards decreased infarct volumes exists in animals treated with MTFL_457_ 1 h after injury, better results are obtained for earlier peptide administration. The therapeutic window for each peptide is likely determined by the specifically targeted mechanism, in this case, TrkB-FL degradation, which is nearly complete 3 h after damage initiation [8]. In contrast, a different peptide, targeting specific protein interactions established by the truncated TrkB-T1 receptor in excitotoxicity and stroke, better modulates the inflammatory response and neurotoxicity when administered 1 h post-injury [67]. A *post-hoc* metanalysis of data from three phase 3 nerinetide trials also recently established the importance of early peptide intervention for penumbra preservation before recanalization therapies [4, 5, 68]. MTFL_457_ could be an alternative cerebroprotective agent used as an adjunct to reperfusion or, additionally, as an enhancer of therapies for neurological and psychiatric diseases based on the use of BDNF or small size analogues [24], which are challenged by TrkB-FL downregulation and GA damage induced by excitotoxicity.

## Materials and methods

### Experimental models

Animal procedures followed European Union Directive 2010/63/EU and were approved by the ethics committees of CSIC and Comunidad de Madrid (Ref PROEX 276.6/20). The housing facilities, approved by Comunidad de Madrid (# ES 280790000188), complied with official regulations. All efforts were undertaken to minimize animal suffering and reduce the number of animals used; all animals maintained a standard health and immune status and were supervised daily by professional caretakers. Upon arrival, male mice were allowed one week to acclimatize to the housing facility. They were kept in groups of fewer than six in standard individually ventilated cages containing bedding and nesting material. One or two pregnant rats were housed similarly in standard cages with bedding and nesting material. Animals were maintained under controlled conditions, including a 12-hour light cycle, regulated relative humidity and temperature, and had *ad libitum* access to irradiated food and water.

### Mouse model of ischemia by photothrombosis

Permanent focal ischemia was induced in the cerebral cortex of adult male *Balb/cOlaHsd mice* (25-30 g; 8-12 weeks of age; Envigo RMS Spain SL) by microvascular photothrombosis as described previously [8]. This model mimics small artery occlusion seen in human stroke, causing focal brain damage with histological and MRI correlations to human patterns [35]. Brain injury involves damage to vascular endothelium, platelet activation, followed by microvascular thrombotic occlusion of a particular region [43], selected for irradiation using a stereotaxic frame. In these experiments, we used coordinates +0.2 AP, +2 ML relative to Bregma to damage the primary motor (hindlimb and forelimb) and somatosensory cortex according to the Paxinos mouse brain atlas. Thus, mice anesthetized with isoflurane (5% for induction, 2% for maintenance in oxygen; Abbot Laboratories, Madrid, Spain) were placed in a stereotaxic frame (Narishige Group, Tokyo, Japan); body temperature was maintained at 36–37 °C with a self-regulating heating blanket (Cibertec, Madrid, Spain). A midline scalp incision exposed the skull and identified Bregma and Lambda points. Then, we used a micromanipulator to center a cold-light (Schott KL 2500 LCD, Schott Glass, Mainz, Germany) with a 1.5 mm diameter fiber optic bundle at the indicated coordinates (right side). Next, the photosensitive dye Rose Bengal (20 mg/kg; Cat#R3877, Sigma-Aldrich) was administered by retro-orbital injection of the venous sinus, followed after 5 min by brain illumination through the intact skull for 10 min (600 lms, 3000 K), causing local dye activation and damage in areas underneath the selected stereotaxic position. Following the surgical procedure, the incision was sutured and mice were allowed to recover. Buprenorphine (0.1 mg/kg) was applied for post-surgical pain relief every 6–12 h.

A single dose (10 nmol/g) of peptides MTMyc (Ac-YGRKKRRQRRRAEEQKLISEEDLLR-NH_2_), MTFL_457_ (Ac-YGRKKRRQRRRHSKFGMKGPASVIS-NH_2_) or MTFL_457_AAA (Ac-YGRKKRRQRRRHSAAAMKGPASVIS-NH_2_) ( > 95% purity; GenScript) were retro-orbitally injected 10 min or 1 h after damage initiation, as indicated. These peptides are N-ter acetylated and C-ter amidated to improve plasma stability. They were prepared as 2.5 mM solutions in 0.9% NaCl and, just before injection, acidity from trifluoroacetic acid counterions in the peptide preparations was neutralized by adding fresh HCO_3_NH_4_ to a final concentration of 44 mM. Mice were not subjected to other procedures before ischemia and were naïve to drug or peptide treatment. Animals were randomly allocated to the experimental groups and the researchers were blinded to group allocation and treatment. For immunohistochemistry, mice deeply anesthetized as before 5 h after damage were intracardially perfused with cold PBS and 4% paraformaldehyde in PBS. Brain processing proceeded as explained below. For assessment of infarct volume, animals were sacrificed by CO_2_ inhalation followed by cervical dislocation 24 or 72 h after damage induction, as indicated. Their brains were sectioned into serial 1-mm-thick coronal slices using a mouse brain matrix (Stoelting, Wood Dale, IL, USA). Slices were stained with 2% TTC (Cat#T8877, Sigma-Aldrich) at room temperature, and fixed in 4% paraformaldehyde before scanning of both rostral and caudal sides. A pre-established exclusion criterion was an infarct volume lower than 2%, considered as a model failure which is probably due to incorrect Rose Bengal administration or failed cold-light illumination. Only one animal was excluded due to this reason. Out of 96 animals, five died during surgery and two immediately after blind peptide administration. For the duration of the in vivo experiments (up to 72 h), no adverse effects were observed and no animals required an early sacrifice.

### Primary culture of rat cortical neurons and treatment

Primary neuronal cultures were obtained from the brain cortex of 18-day-old *Wistar rat embryos* (E18), using both genders indistinctly, as previously described [9]. Briefly, dissected cortices were mechanically dissociated in Minimum Essential Medium supplemented with 22.2 mM glucose, 0.1 mM GlutaMAX (Cat#35050-038, Gibco), 5% fetal bovine serum (Cat#FBS-HI-12A, Capricorn), 5% donor horse serum (Cat#11510516; Life Technologies), and 100 U/ml penicillin/100 µg/ml streptomycin (Cat#15140148; Life Technologies). Then, the cell suspension was seeded at 1×10^6^ cells/ml in the same medium onto plates previously treated overnight at 37 °C with poly-L-lysine (100 µg/ml; Cat#P1524, Sigma-Aldrich) and laminin (4 µg/ml; Cat#L2020, Sigma-Aldrich). In these mixed cultures, early growth of the glial subpopulation helps maintain non-toxic glutamate levels during neuronal maturation. After 7 days in vitro (DIV), further glial proliferation was inhibited using cytosine β-D-arabinofuranoside (AraC, 10 µM; Cat#C1768, Sigma-Aldrich), and growth continued until 13 DIV. Mature neurons were then either subjected to excitotoxicity as indicated below or treated with BDNF (100 ng/ml; Cat#450-02, PeproTech) for the specified times. When indicated, cultures were preincubated with MTMyc, MTFL_457_, or MTFL_457_AAA (25 µM, 30 min) before excitotoxicity induction, with peptides remaining in the culture media throughout the experiment. Mature neurons were also treated for 30 min with dynasore hydrate (80 μM; Cat#D7693, Sigma-Aldrich) before NMDA addition as indicated.

### Induction of neuronal excitotoxicity and evaluation of neuronal viability

Cultures were incubated for the indicated times with NMDA (100 µM; Cat#0114, Tocris) and its co-agonist glycine (10 µM; Cat#161-0718; Bio-Rad), hereafter referred to as NMDA, to induce a strong excitotoxic response in mature neurons within the mixed culture, without affecting astrocyte viability as previously described [69]. To analyze shedding of TrkB fragments via MP action, culture medium was collected before cell lysis for immunoblot analysis. Neuronal viability was measured by the MTT reduction assay. MTT (0.5 mg/ml; Cat#M5655, Sigma-Aldrich) was added to culture medium 2 h after NMDA treatment, and incubation continued for an additional 2 h at 37 °C. Produced formazan salts were then solubilized in DMSO and quantified spectrophotometrically at 570 nm. To establish the contribution of quiescent glial cells to viability in the mixed primary cultures, sister cultures were treated with 400 μM NMDA and 10 μM glycine 24 h before the MTT assay. These conditions cause mature neuron death but no glial damage [69]. The glial contribution to total cell viability was then subtracted to calculate the viability of the neuronal subpopulation in each sample. Each experiment included sample triplicates per treatment, and multiple independent experiments were performed as detailed in the figure legends. The viability of neurons preincubated with a peptide and subjected to excitotoxicity was calculated relative to that of neurons preincubated with the same peptide but without NMDA treatment.

### Western blot analysis

Cultured cells were lysed in RIPA buffer (50 mM Tris-HCl pH 8, 150 mM NaCl, 1% sodium deoxycholate, 1% NP-40, 1 mM DTT, 0.1% SDS) containing protease and phosphatase inhibitors (Complete protease and PhosSTOP inhibitor cocktails, Roche; Cat#11 697 498 001, Cat#04 906 837 001). Protein concentration was determined using the BCA Protein Assay Kit (Thermo Fisher; Cat#A 2001), and lysates were denatured in Laemmli SDS-sample buffer by heating at 95 °C for 5 min. Equal amounts of total cell lysates were resolved by Tris-Glycine SDS-PAGE and transferred onto Protran nitrocellulose membranes (GE Healthcare; Cat#GE10600002). Protein transfer efficacy was confirmed by Ponceau S staining (1% w/v). After blocking with 5% non-fat dry milk in Tris-buffered saline (TBS) containing 0.05% Tween-20 (TBS-T), membranes were incubated overnight at 4 °C with primary antibodies, washed, and then incubated with appropriate HRP-conjugated anti-rabbit (Bethyl; Cat#A120-108P, RRID:AB_10892625) or anti-mouse (Bethyl; Cat#A90-137P, RRID:AB_1211460) secondary antibodies for 1 h at room temperature. Finally, immunoreactivity was detected using Clarity Western ECL Substrate (BioRad; Cat#1705060), and band intensity was quantified via densitometry (Adobe Photoshop). Protein levels were normalized to neuron-specific enolase (NSE) in the same sample and expressed relative to their respective controls (set to 100%). This neuronal loading control was chosen since NSE levels are unaffected by NMDA treatment. Conversely, calpain activation induced by excitotoxicity was confirmed by analyzing characteristic brain spectrin breakdown products (BDPs; 150 and 145 kDa), a standard substrate of this protease. Multiple independent experiments were performed and quantified as detailed in the figure legends. Primary antibodies against the following proteins were used: Hrs (Santa Cruz Biotechnology; Cat#sc-271455, RRID:AB_10648901), NSE (Millipore; Cat#AB951, RRID:AB_92390), spectrin alpha chain (Millipore; Cat#MAB1622, RRID:AB_94295), TrkB-FL C-ter (Santa Cruz Biotechnology; sc-11, RRID:AB_632554), TrkB extracellular sequences or panTrkB (Santa Cruz Biotechnology; sc-136990, RRID:AB_2155262), PSD-95 C-ter (Transduction Laboratories; Cat#610496, RRID:AB_2315218), pY816-TrkB-FL (Boster; Cat#P01388). Full and uncropped Western blots corresponding to Figs. 1, 3, 4, S2 and S3 are provided as ‘Supplemental Material’.

### Protein immunoprecipitation

Total cell lysates were prepared from cortical cultures in 1% NP-40, 80 mM NaCl, 20 mM EDTA, and 20 mM Tris-HCl (pH 8), containing protease and phosphatase inhibitors as described above. Approximately 0.3-1 mg of cleared lysate was incubated for 1 h at 4 °C with 3-4 µg of the indicated antibody before adding 60 µl of 50% Protein G Agarose (Thermo Fisher; Cat#15920010) and incubating for 1 h at room temperature with continuous shaking. Subsequently, equivalent volumes of the immunoprecipitated complexes were analyzed by immunoblotting alongside equal amounts of the starting total lysates, as indicated.

### Biotinylation of cell-surface proteins

Following 1 h NMDA treatment, cultures were immediately washed with ice-cold PBS containing 1 mM CaCl_2_ and 0.5 mM MgCl_2_. Surface proteins were then biotinylated for 30 min at 4 °C using 0.5 mg/ml EZ-Link Sulfo-NHS-SS-biotin (Thermo Fisher; Cat#21331) prepared in the same buffer. Excess free biotin was removed by washing cultures with cold PBS/CaCl_2_/MgCl_2_ containing 0.1% BSA, followed by two additional washes with the same buffer lacking BSA. Next, cells were lysed in RIPA buffer (containing protease and phosphatase inhibitors but without DTT), saving a small aliquot of the total extract for input analysis. The remaining total extracts were incubated with streptavidin resin (GenScript; Cat#L00353) for 3 h at 4 °C to precipitate biotinylated proteins. Streptavidin-biotin complexes were washed twice with lysis buffer containing 500 mM NaCl (plus inhibitors) and twice with lysis buffer omitting this salt (plus inhibitors). Pellets were then solubilized and denatured in SDS-PAGE sample buffer (10 min, 50 °C). Equivalent volumes of the isolated protein fraction and total protein extracts were analyzed in parallel by Western blot. Multiple independent experiments were performed and quantified as detailed in the figure legends.

### Immunocytochemistry

Primary cultures, grown as before on coverslips coated with poly-L-lysine/laminin, were treated as indicated and fixed with 4% paraformaldehyde in PBS for 30 min. After washing with PBS, cells were blocked and permeabilized for 30 min at room temperature using 1% BSA and 0.1% Triton X-100 in PBS. Coverslips were then incubated overnight at 4 °C with primary antibodies diluted in the same buffer, recognizing: Hrs (Santa Cruz Biotechnology; Cat#sc-271455, RRID:AB_10648901), TrkB-FL C-ter (Santa Cruz Biotechnology; sc-11, RRID:AB_632554), and GM130 (BD Biosciences; Cat# A-610822, RRID:AB_398141). Detection was achieved using appropriate goat secondary antibodies conjugated to Alexa Fluor 488 (Thermo Fisher Scientific; anti-rabbit, Cat #A-11034, RRID:AB_2576217; anti-mouse, Cat#A-11029, RRID:AB_2534088) or Alexa Fluor 546 (Thermo Fisher Scientific; anti-rabbit, Cat#A-11035, RRID:AB_2534093), diluted similarly. Afterwards, coverslips were incubated for 10 min with DAPI (0.5 µg/ml; Molecular Probes, Cat#D1306) before mounting with Prolong Diamond antifade reagent (Molecular Probes, Cat#P36970). Confocal images (single sections) were acquired using a Zeiss LSM 710 inverted laser confocal microscope (Jena, Germany; 63x Plan-Apochromatic oil objective) and normalized per channel. Images were processed for presentation with ImageJ (NIH).

For colocalization studies, single optical sections from z-series were selected, with each channel acquired sequentially using confocal microscopy as above. For each condition, equivalent sections were chosen, and a minimum of 80 neurons were analyzed. We calculated the Pearson correlation coefficient (PCC) and the percentage of cells with PCC values ≥ 0.50 (or the indicated threshold). Four independent experiments were quantified. To analyze Golgi apparatus (GA) fragmentation, we used the Fiji image processing package (https://www.nature.com/articles/nmeth.2019; RRID:SCR_002285) and a macro established by Dr. Sánchez-Ruiloba (Instituto de Investigaciones Marinas, CSIC). All images were analyzed similarly. From the optical sections acquired, we selected a central one where labeled particles were best detected. After setting a threshold to define positive signals, particles were identified, and their area, mean intensity, circularity, and integrated density were obtained. We analyzed 3-5 images per condition in each experiment and calculated mean values from 5 independent experiments. Representative images fitting the final mean values are shown to represent particle values for specific conditions.

### Measurement of infarct volume

Rostral and caudal images of coronal slices stained with TTC (as described above) were analyzed using ImageJ software (https://imagej.net/; RRID:SCR_003070) by an observer blinded to experimental groups. After image calibration, the delineated areas of the ipsilateral and contralateral hemispheres, and the infarcted region (unstained area), were measured. Considering slice thickness, the corresponding volumes were calculated and then corrected for edema effects, estimated by comparing total hemisphere volumes. The corrected infarct volumes were expressed as a percentage relative to the contralateral hemisphere to account for normal brain size differences between animals. For each animal, the mean result from the rostral and caudal slice faces was calculated.

### Immunohistochemistry and Fluoro-Jade C staining

Brains obtained from animals sacrificed 5 h after injury were post-fixed in 4% paraformaldehyde in PBS at 4 °C for 24 h and cryoprotected by immersion in 30% sucrose for 48 h at 4 °C. Coronal frozen sections (30 μm thick) were prepared using a cryostat (Leica, Heidelberg, Germany). Sections were incubated in flotation with blocking solution (10% goat serum, 0.5% Triton X-100 in PB) for 1 h at room temperature and then overnight at 4 °C with mouse anti-GM130 antibody (Cat# A-610822, RRID:AB_398141, BD Biosciences), followed by incubation for 2 h at room temperature with Alexa Fluor 546-conjugated goat anti-mouse secondary antibodies (Cat#A-11030, RRID:AB_2534089, Thermo Fisher Scientific) and DAPI (5 µg/ml; Cat#D1306, Molecular Probes) for nuclear staining. After washing in distilled water, sections were mounted on slides, dried overnight at RT, cleared in xylene, and cover-slipped with DPX (Cat#06522, Sigma-Aldrich). For control experiments, incubation with the anti-GM130 antibody was omitted. In indicated experiments, an additional step was added after blocking: sections were incubated for 18 h at 4 °C with a goat anti-mouse IgG H&L antibody (Fab fragment; Cat#ab6668, RRID:AB_955960, Abcam) diluted in 5% goat serum, 0.5% Triton X-100 in PB, before washing and incubation with the secondary antibody. This step minimized background staining caused by reaction of anti-mouse secondary antibodies with endogenous mouse immunoglobulins that had leaked into the ischemic area due to BBB disruption [46].

For combined double immunohistochemistry and Fluoro-Jade C staining, after blocking, brain sections were incubated overnight at 4°C with mouse anti-GM130 and rabbit anti-SNTF antibodies (Cat#ABN2264, Millipore), followed by incubation for 2 h at room temperature with goat Alexa Fluor 546-conjugated anti-mouse (Cat#A-11030; RRID:AB_2534089, Thermo Fisher Scientific) and Alexa Fluor 647-conjugated anti-rabbit secondary antibodies (Cat#A-21245, RRID:AB_2535813, Thermo Fisher Scientific). Sections were then rinsed in distilled water and incubated with a solution of 0.0004% Fluoro-Jade C (Cat#AG325, Millipore) and 0.0002% DAPI in 0.01% acetic acid for 10 min at room temperature to specifically label degenerating neurons in the infarcted area and cell nuclei, respectively. Confocal images were acquired with a 63x Plan-Apochromatic oil immersion objective and processed for presentation as described above.

### Quantification and statistical analysis

All data were expressed as mean ± standard deviation (SD) from 3 to 9 independent experiments. Detailed information about experiment numbers, sample sizes, and specific statistical tests for each analysis are provided in the figure legends. Cell samples used in individual experiments were sister primary cultures grown in multiwell plates, derived from the same cell suspension, with treatments assigned randomly. For the evaluation of neuronal viability, triplicates were analyzed per treatment condition in each individual experiment. Using totally independent cell suspensions, prepared from different pregnant rats, individual experiments were replicated as indicated by the n number. There was no previous estimation of sample size in the case of the in vitro experiments. For the in vivo neuroprotection study, the number of required animals was calculated using G*Power 3.1.9.7 software, with a statistical power of 0.8 and an alpha error probability of 0.05. Statistical analyses were performed using the Statistical Package for Social Science (SPSS, v.18, RRID:SCR_002865, IBM) and GraphPad Prism 8.4.3.686 (https://www.graphpad.com; RRID:SCR_002798). As a previously established criteria, we calculated outliers with GraphPad, excluding values more than three standard deviations outside the mean. Based on the number of experimental groups, data distribution (Kolmogorov-Smirnov and Shapiro-Wilk’s tests), and homogeneity of variances (Levene’s test), we applied unpaired Student’s *t*-test, one-way or two-way ANOVA followed by Bonferroni test, or a generalized linear model followed by Fisher’s LSD *post-hoc* test. A P value less than 0.05 was considered statistically significant (^*^P < 0.05, **P < 0.01, ^***^ *P* < 0.001).

## Supplementary information

Full and uncropped Western blots

Supplementary Figures S1-S6

## Data Availability

All data supporting the conclusions of this article are included within the article and in the additional file provided.
