# Correction: Retrograde transport of neurotrophin receptor TrkB-FL induced by excitotoxicity regulates Golgi stability and is a target for stroke neuroprotection

**DOI:** 10.1038/s41419-026-08785-z

**Published:** 2026-05-05

**Authors:** Gema María Esteban-Ortega, Elena Torres-Campos, Margarita Díaz-Guerra

**Affiliations:** 1 Instituto de Investigaciones Biomédicas Sols-Morreale (IIBM), Consejo Superior de Investigaciones Científicas-Universidad Autónoma de Madrid, Madrid, 28029 Spain; 2 Present Address: Centro de Biología Molecular Severo Ochoa (CBMSO), Consejo Superior de Investigaciones Científicas-Universidad Autónoma de Madrid, Nicolás Cabrera,1, Madrid, 28049 Spain

**Keywords:** Stroke, Stroke

Correction to: *Cell Death & Disease* 10.1038/s41419-025-07990-6, published online 29 August 2025

We inadvertently duplicated one image of Figure 5D and, thus, the photo representing cultures pre-incubated with peptide MTFL457 treated for 60 min with NMDA is not correct. Our correction does not affect the conclusions of this article. We apologize for the mistake and any inconvenience caused.

**Original Figure 5** 
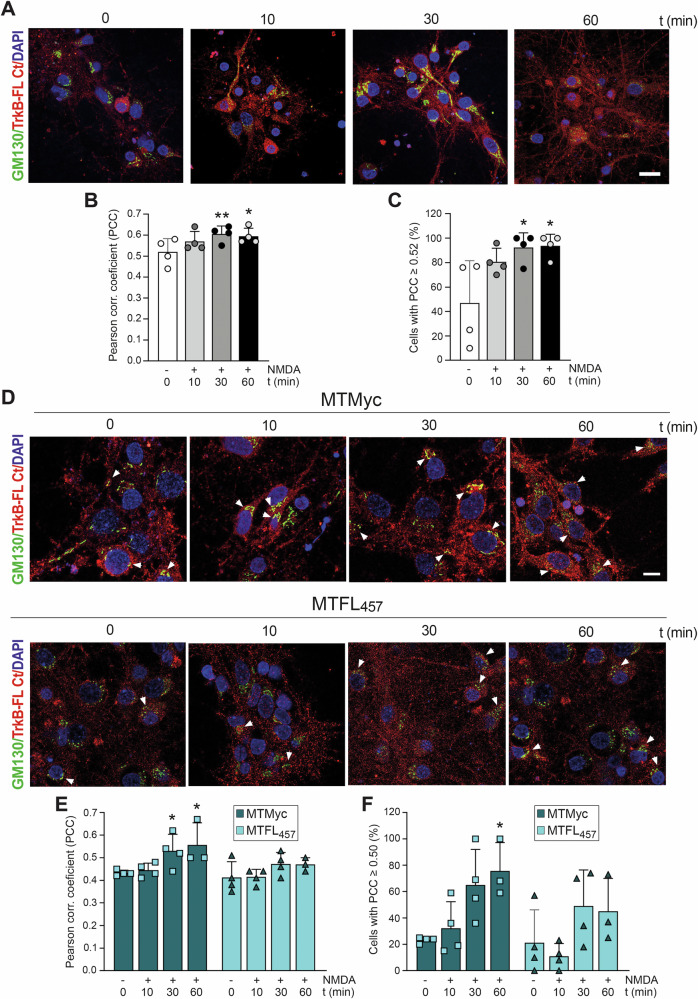


**Amended Figure 5** 
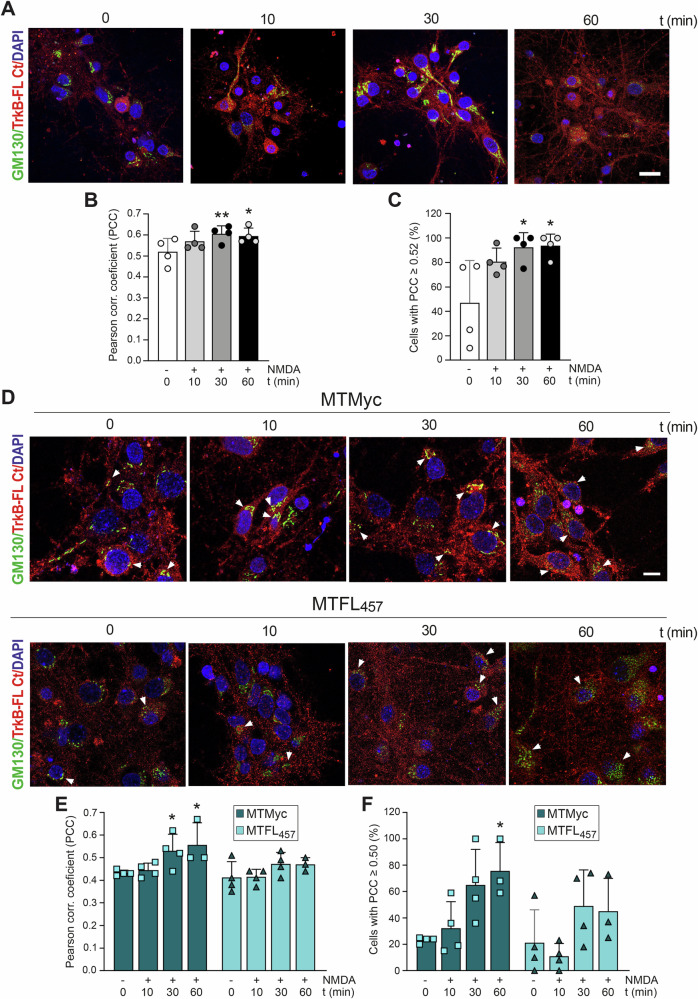


The original article has been corrected.

