# Supplementary material for: Retrograde transport of neurotrophin receptor TrkB-FL induced by excitotoxicity regulates Golgi stability and is a target for stroke neuroprotection
Source: Cell Death Dis. 2025 Aug 29;16(1):659. doi: 10.1038/s41419-025-07990-6 (PMC12397353; doi:10.1038/s41419-025-07990-6)

Full-length blots Fig.1

Fig. 1B

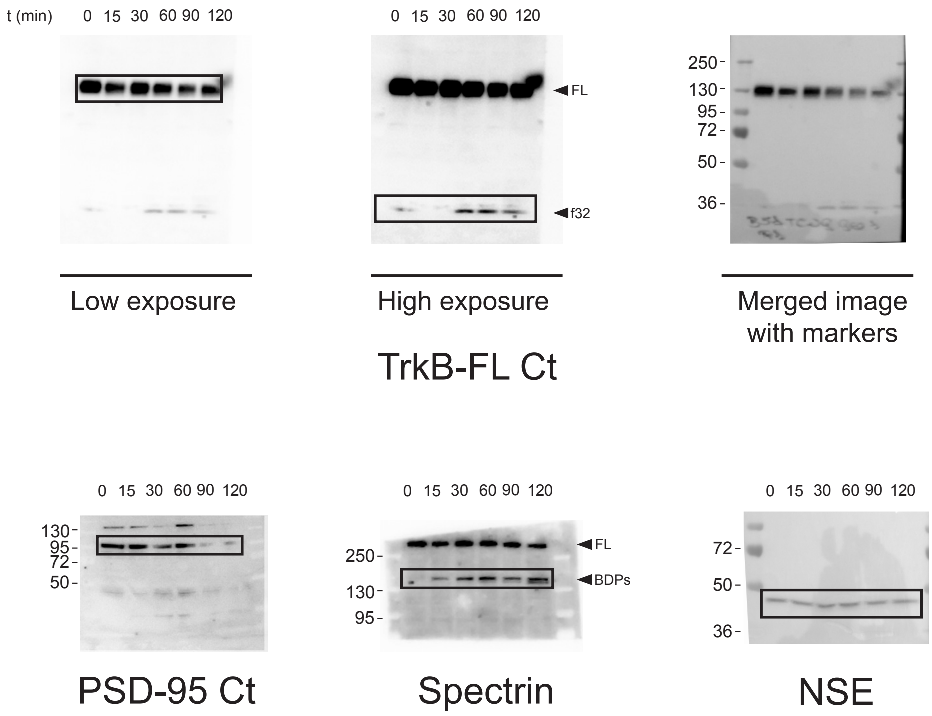

Fig. 1E

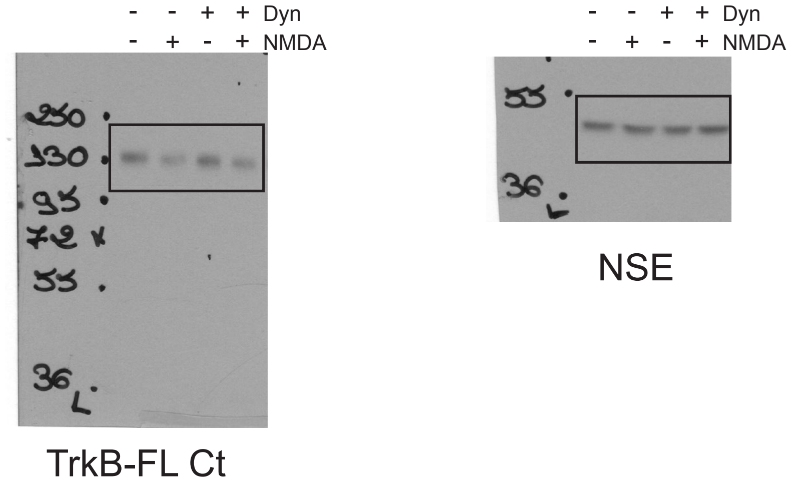

Fig. 1H

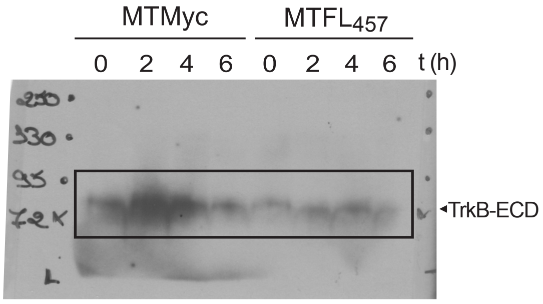

panTrkB

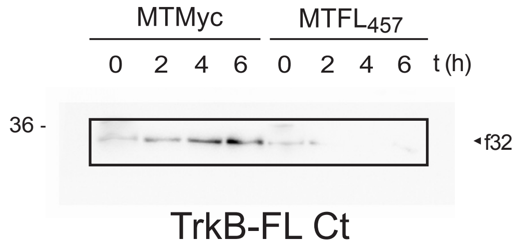

TrkB-FL Ct

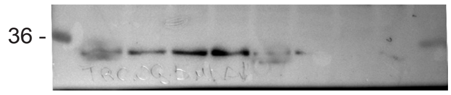

Merged image with markers

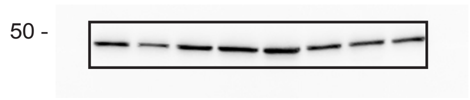

NSE

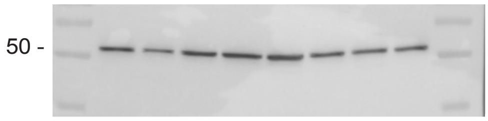

Merged image with markers

Fig. 1J

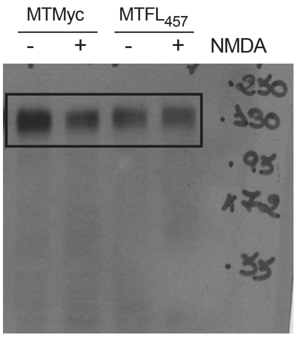

pY816TrkB-FL

Fig. 1K

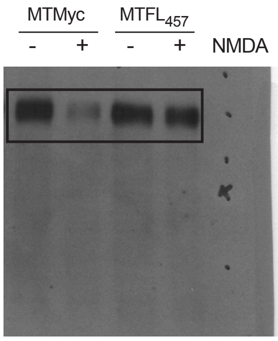

pY816TrkB-FL

Full-length blots Fig.3

Fig. 3D

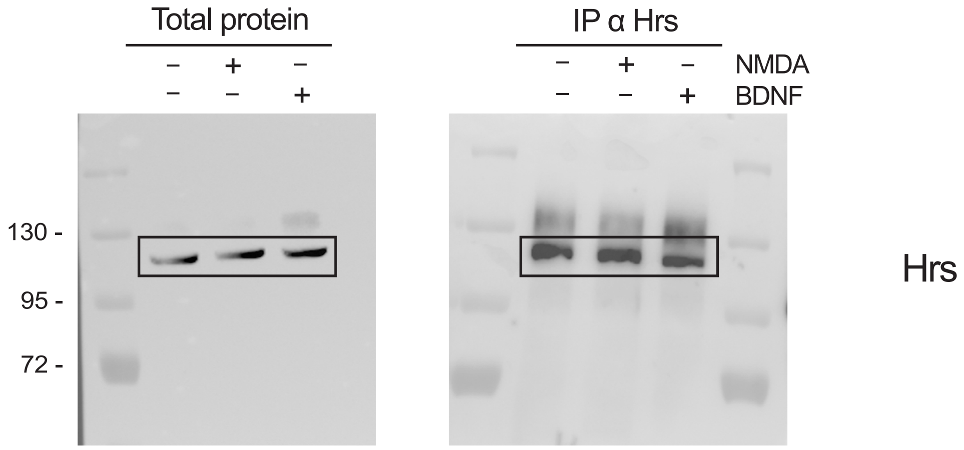

Fig. 3E

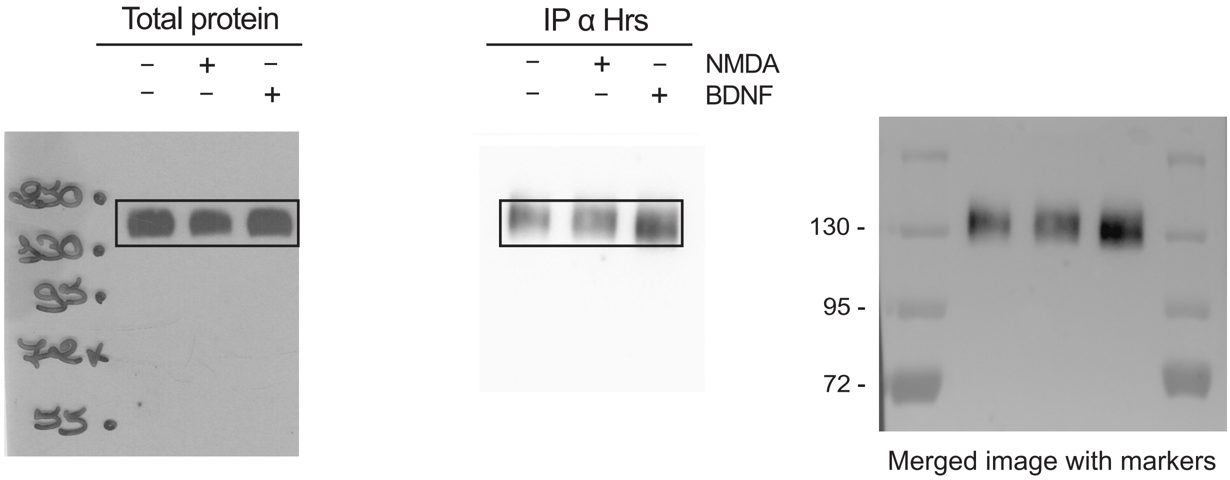

TrkB-FL Ct

Full-length blots Fig. 4

Fig. 4C

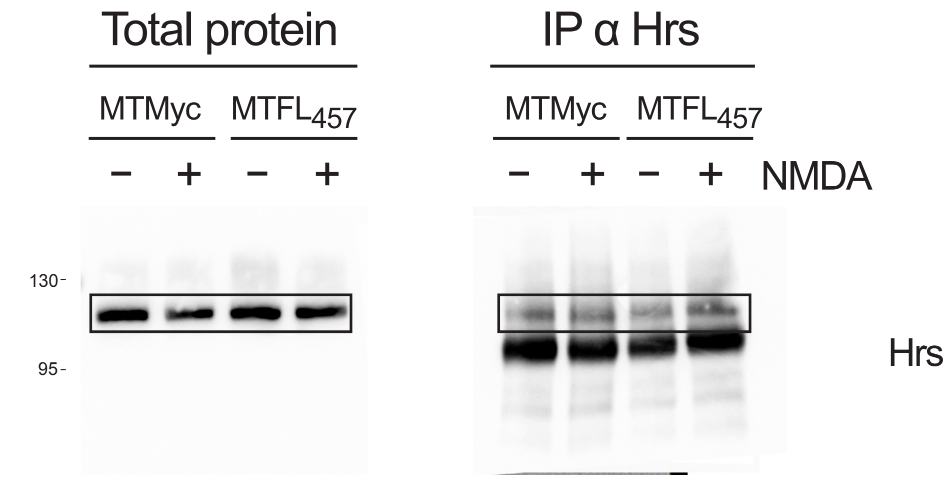

Fig. 4D

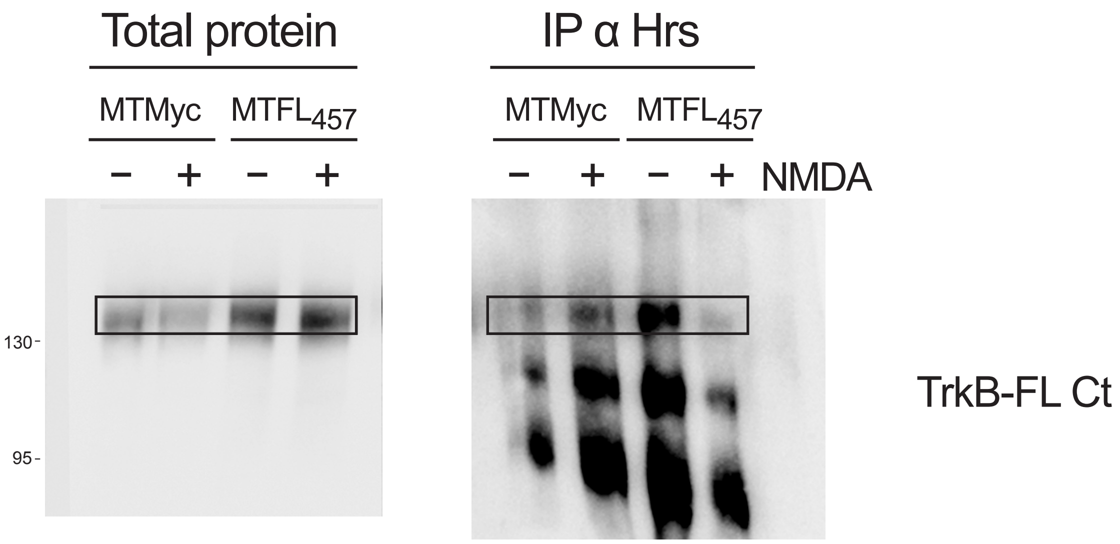

Full-length blots Fig. S2

Fig. S2A

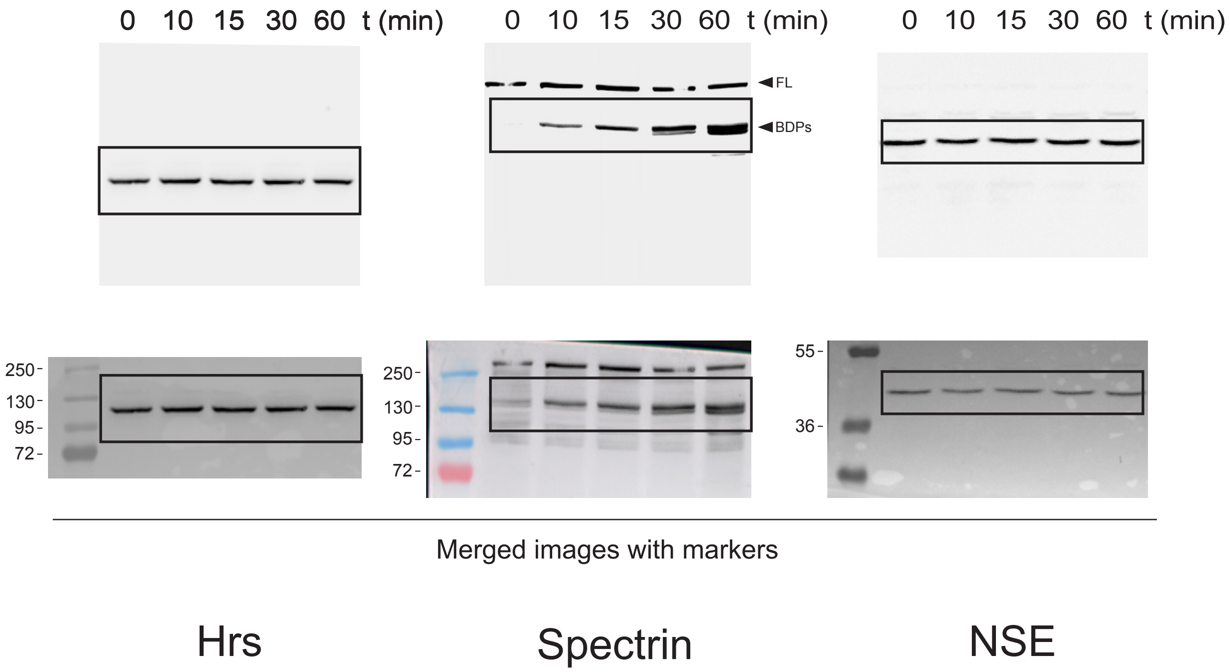

Fig. S2B

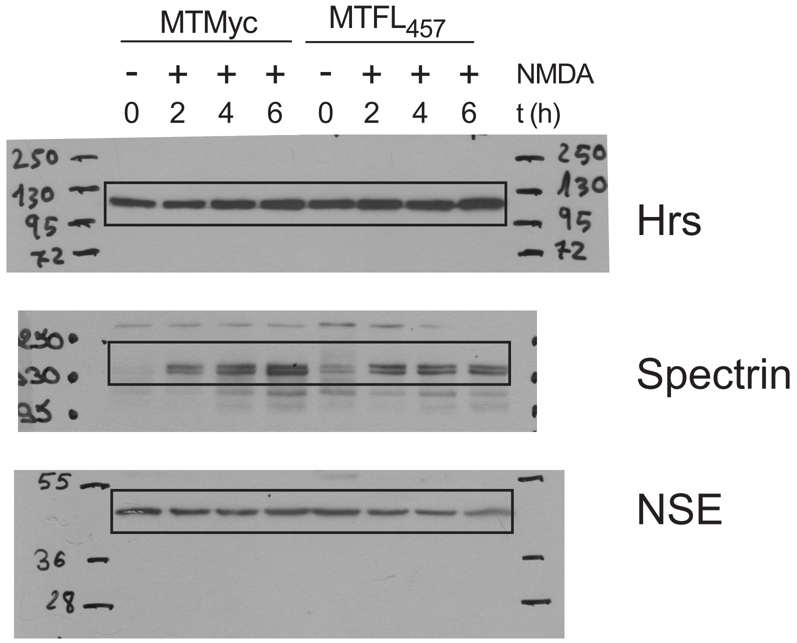

Full-length blots Fig. S3

Fig. S3A

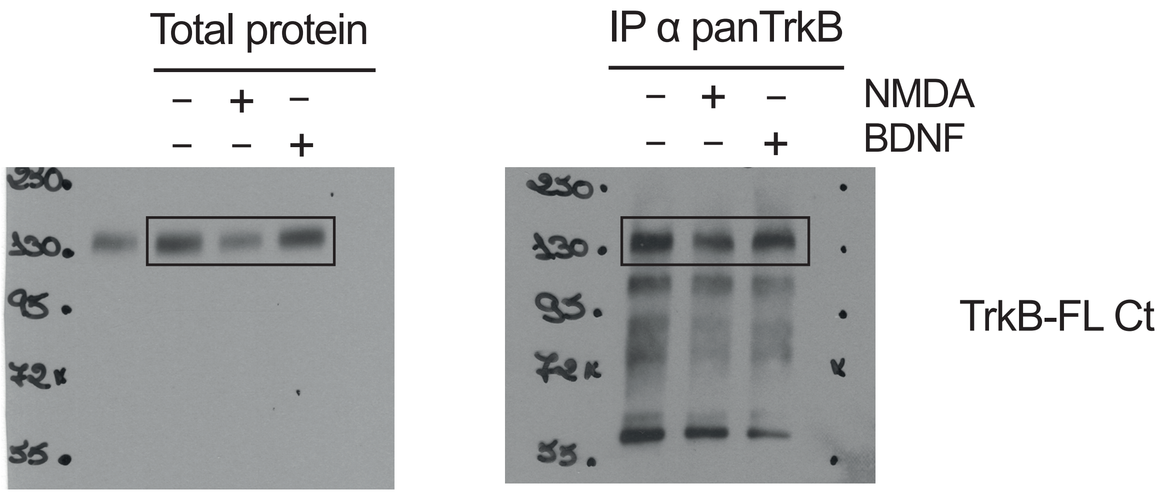

Fig. S3B

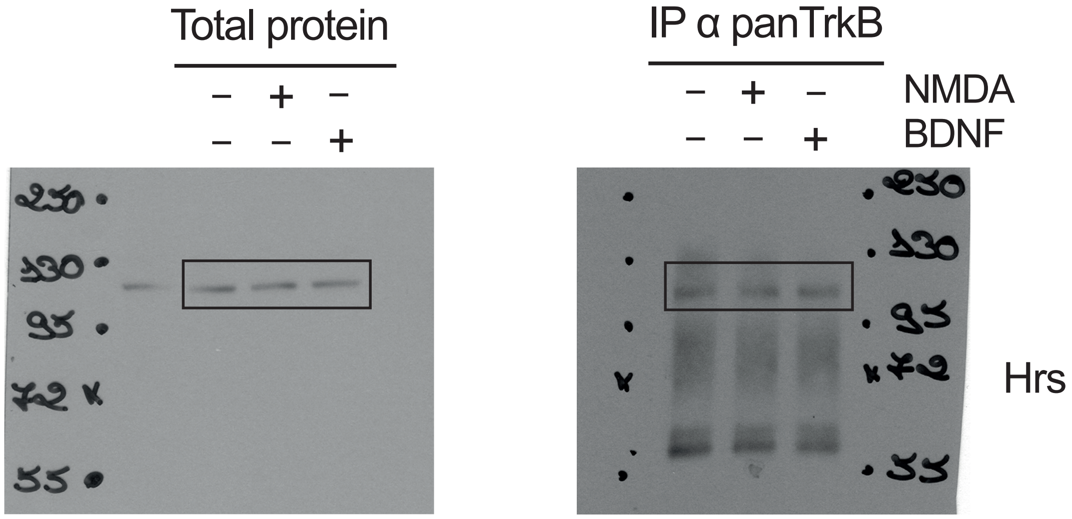

Supplement: Supplementary file 1 — Full and uncropped Western blots [file 41419_2025_7990_MOESM1_ESM.pdf]
