# Supplementary material for: Retrograde transport of neurotrophin receptor TrkB-FL induced by excitotoxicity regulates Golgi stability and is a target for stroke neuroprotection
Source: Cell Death Dis. 2025 Aug 29;16(1):659. doi: 10.1038/s41419-025-07990-6 (PMC12397353; doi:10.1038/s41419-025-07990-6)
Supplement: Supplementary file 2 — Supplementary Figures S1-S6 [file 41419_2025_7990_MOESM2_ESM.pdf]

Supplementary Information

Retrograde transport of neurotrophin receptor TrkB-FL induced by excitotoxicity regulates Golgi stability and is a target for stroke neuroprotection

Gema María Esteban-Ortega[1], Elena Torres-Campos[1] and Margarita Díaz-Guerra[1,*]

[1]Instituto de Investigaciones Biomédicas Sols-Morreale (IIBM), Consejo Superior de Investigaciones Científicas-Universidad Autónoma de Madrid, Madrid 28029, Spain

*Corresponding author: mdiazguerra@iib.uam.es

Supplementary Figures

Fig. S1 Effect of peptides $MTFL_{457}$ and $MTFL_{457}AAA$ on infarct volume and neurological outcome in animals treated 1 h after damage initiation.

Fig. S2 Analysis of a possible effect of excitotoxicity on Hrs levels

Fig. S3 Effect of excitotoxicity on TrkB-FL/Hrs coimmunoprecipitation.

Fig. S4 Regulation by peptide $MTFL_{457}$ of excitotoxicity-induced GA fragmentation.

Fig. S5 Leakage of mouse immunoglobulins to the brain cortex due to BBB breakage.

Fig. S6 Leakage of mouse immunoglobulins causes high backgrounds in immunohistochemistry of the ischemic tissue.å

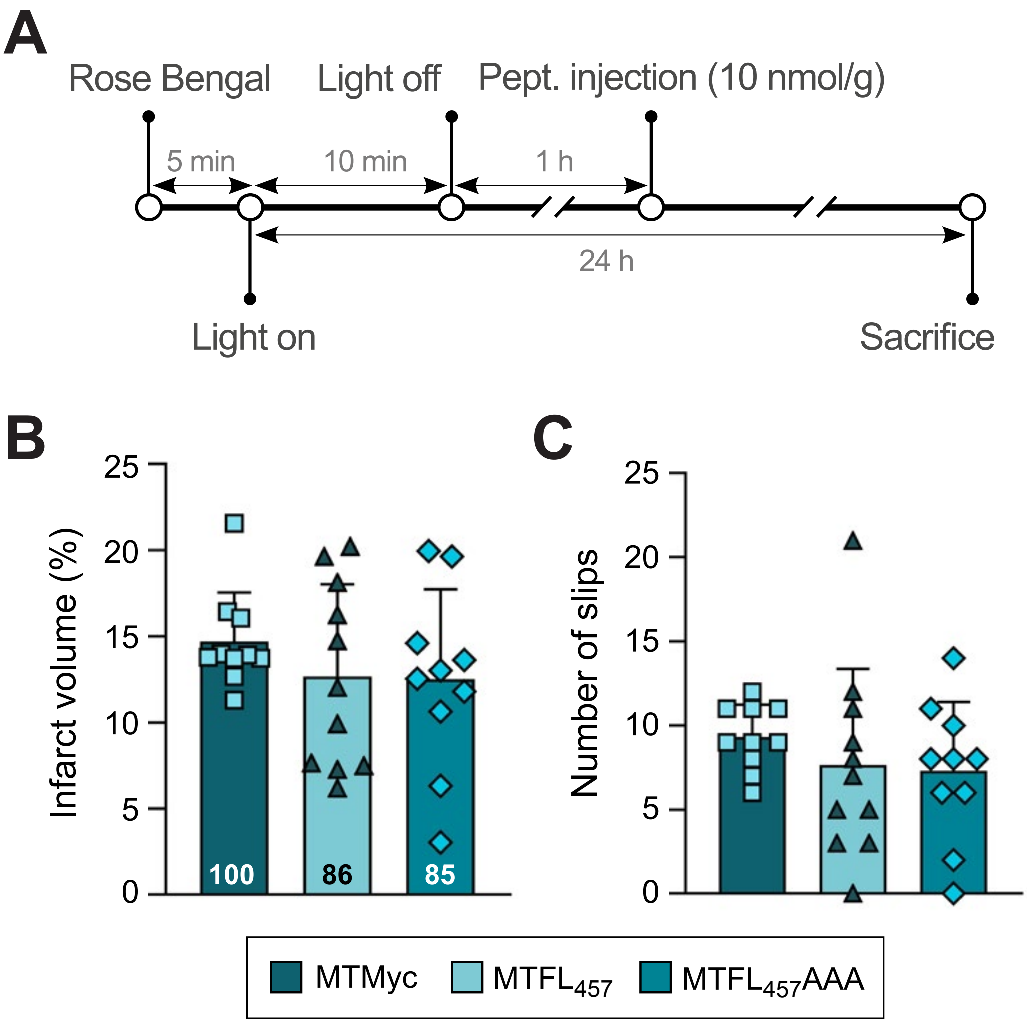

Fig. S1 Effect of peptides MTFL457 and MTFL457AAA on infarct volume and neurological outcome in animals treated 1 h after damage initiation. A Timeline to analyze *in vivo* effects of MTMyc, MTFL457 or MTFL457AAA in the mice model of ischemia. Microvascular photothrombotic permanent damage was initiated by cold-light irradiation (10 min) of a stereotaxically selected brain area after i.v. injection of photosensitive dye Rose Bengal as detailed in Material and Methods. CPPs (10 nmol/g) were retro-orbitally administered 1 h after damage initiation and animals were sacrificed 24 h later. **B** Infarct volume of CPP-injected animals expressed as a percentage of the hemisphere volume. Means ± SD are given (n = 10-11). Infarct volume for MTFL457 and MTFL457AAA experimental groups are also expressed as a percentage of values obtained in animals injected with MTMyc. **C** Evaluation of balance and motor coordination by number of contralateral hind paw slips. Differences with control animals were analyzed by Student's t-test.

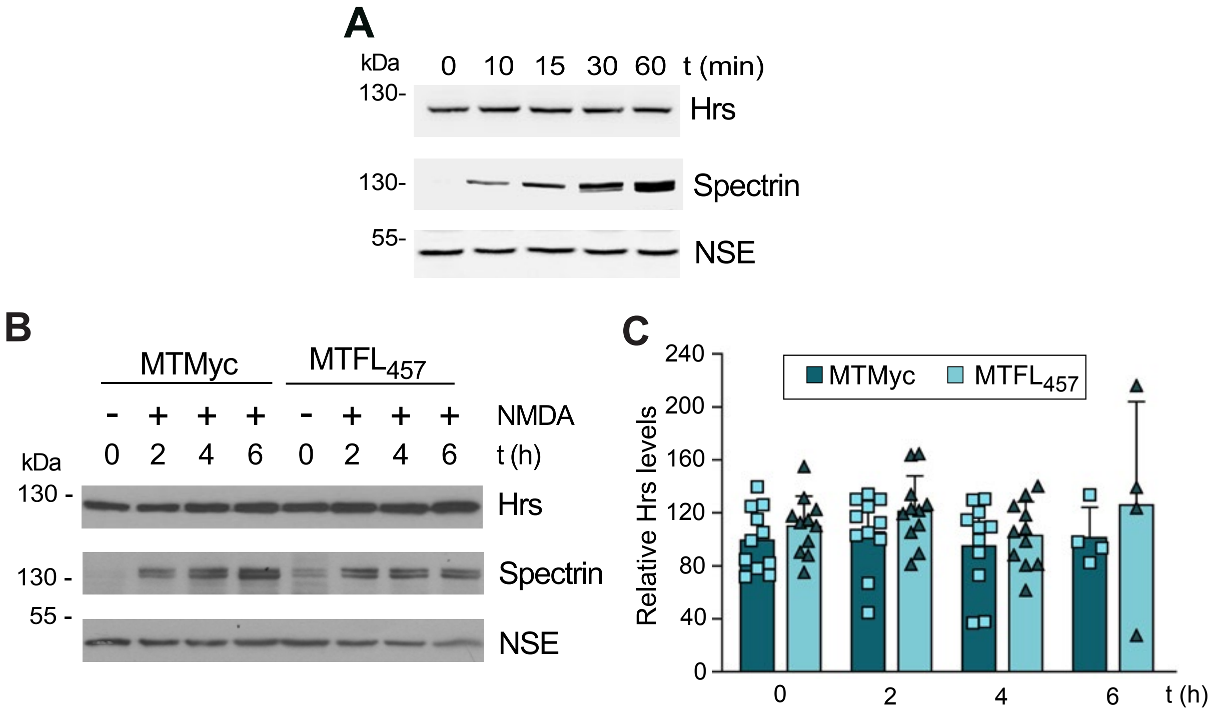

Fig. S2 Analysis of a possible effect of excitotoxicity on Hrs levels. A Cortical neurons were briefly treated with NMDA (0-60 min) and levels of endosomal protein Hrs established by immunoblot. Calpain activation was demonstrated by cleavage of spectrin. **B** Cell cultures were preincubated with MTMyc and $MTFL_{457}$ (25 μM, 30 min) and subsequently treated with NMDA for the indicated times (0-6 h). Levels of Hrs were analyze as above. **C** Mean ± SD (0-4 h, n = 11; 6 h, n = 4) of normalized Hrs levels relative to those obtained in MTMyc preincubated neurons in the absence of NMDA. Statistical analysis was performed using two-way ANOVA followed by a Bonferroni test, no statistically significant differences being found.

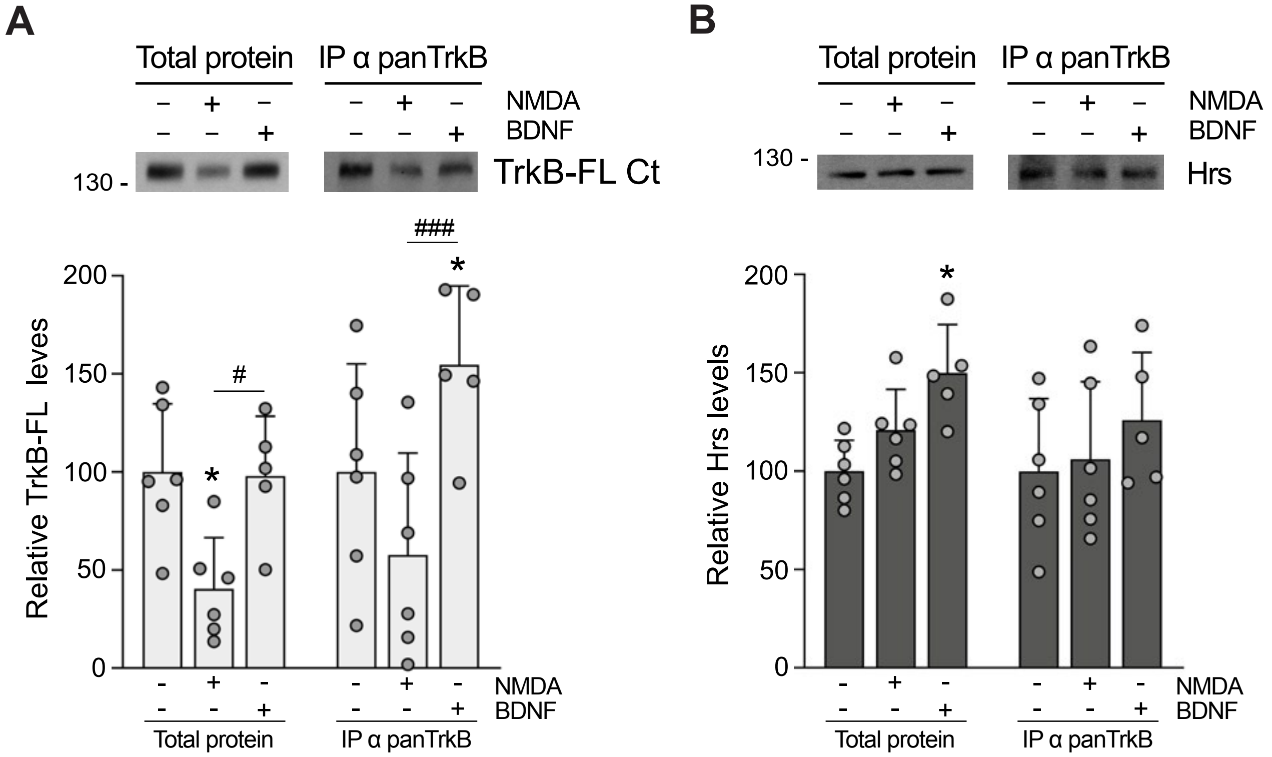

Fig. S3 Effect of excitotoxicity on TrkB-FL/Hrs coimmunoprecipitation. Neuronal cultures were treated with NMDA (100 μM) or BDNF (100 ng/ml) for 30 min and compared to untreated cultures. Proteins immunoprecipitated with antibody panTrkB (IP) were analyzed by immunoblot with the TrkB-FL Ct antibody (**A**) or that recognizing Hrs (**B**) in parallel to the corresponding total protein lysates. Mean values ± SD (n = 6, except for BDNF-treated cells where n = 5) of TrkB-FL and Hrs levels in NMDA or BDNF-treated cultures relative to untreated cells is represented. Statistical analysis was performed using a using a generalized linear model followed by a post-hoc Fisher's LSD test (* $P < 0.05$, relative to the untreated cells; $^{\#} P < 0.05$ and $^{\#\#\#} P < 0.001$ as indicated).

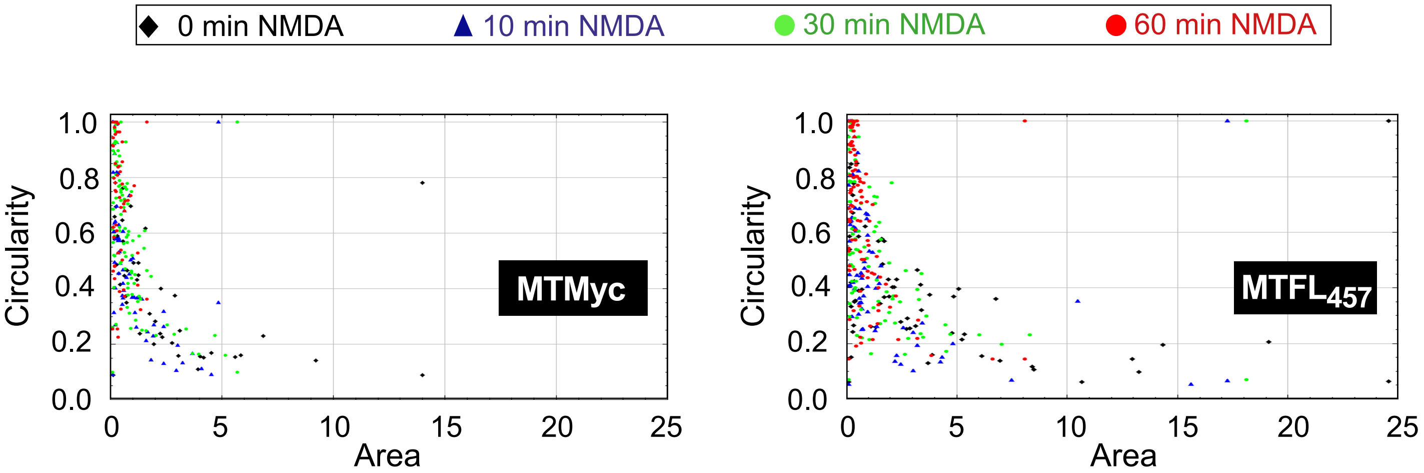

Fig. S4 Regulation by peptide MTFL457 of excitotoxicity-induced GA fragmentation. Cortical neurons preincubated with MTMyc and MTFL457 (25 µM, 30 min) and treated with NMDA for 0-60 min were analyzed by immunofluorescence with the GM130 antibody to visualize GA disruption as described. Representation of area versus circularity for each of the GA particles detected in representative images corresponding to cultures preincubated with MTMyc (left panel) or MTFL457 (right panel), and treated with NMDA for the indicated times. A 0 value indicates absence of circularity, and 1 total circularity.

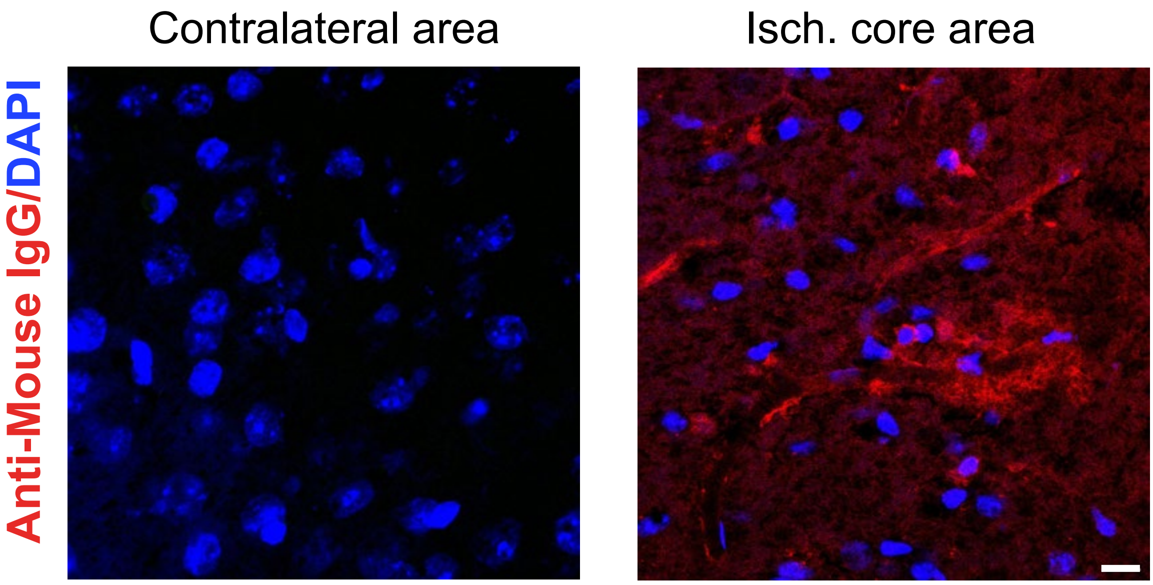

Fig. S5 Leakage of mouse immunoglobulins to the brain cortex due to BBB breakage. Brain coronal sections of animals sacrificed 5 h after insult were analyzed by immunohistochemistry with an anti-mouse secondary antibody, without the primary antibody. Heavy staining of blood vessels and high backgrounds, which challenge GM130 detection, were specifically observed in the ischemic tissue. Representative images correspond to single sections. Scale bar: 10 µm.

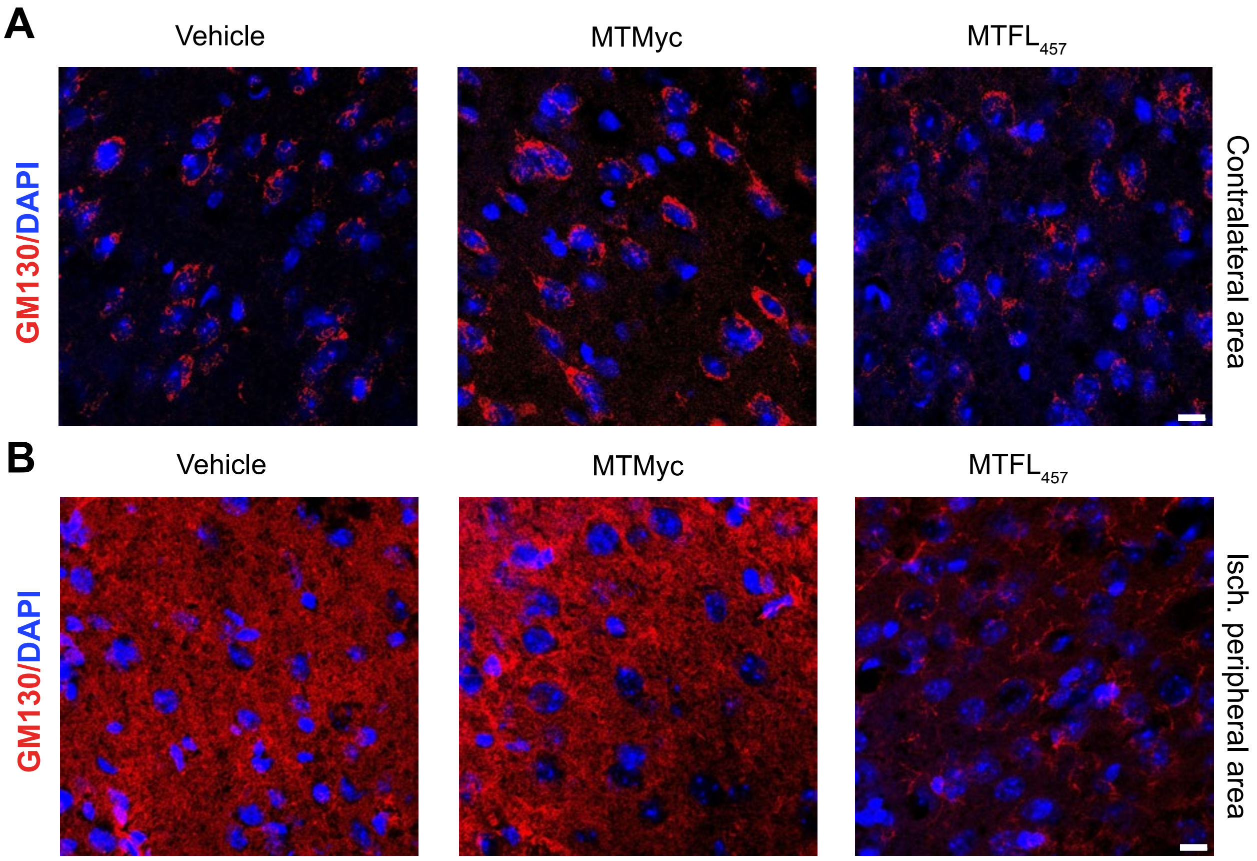

Fig. S6 Leakage of mouse immunoglobulins causes high backgrounds in immunohistochemistry of the ischemic tissue. Animals retro-orbitally injected with peptides MTMyc or $MTFL_{457}$ (10 nmol/g) or vehicle 10 min after damage initiation were sacrificed 5 h later. Comparison of GM130 staining in the contralateral (**A**) or the ischemic peripheral area (**B**) is shown. Representative images correspond to single sections. Scale bar: 10 μm.
